# Terminal Alkyne Activation by an Al(I)-Centered Anion: Impact on the Mechanism of Alkali Metal Identity

**DOI:** 10.1021/acs.organomet.4c00435

**Published:** 2024-12-09

**Authors:** Han-Ying Liu, Henry T. W. Shere, Samuel E. Neale, Michael S. Hill, Mary F. Mahon, Claire L. McMullin

**Affiliations:** Department of Chemistry, University of Bath, Claverton Down, Bath BA2 7AY, U.K.

## Abstract

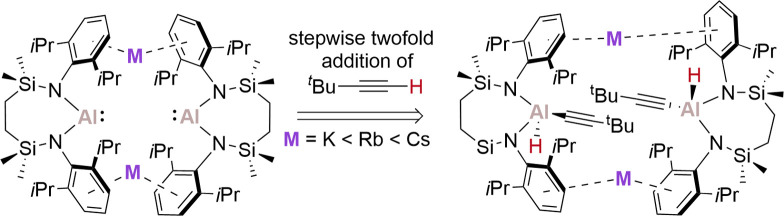

The group 1 alumanyls, [{SiN^Dipp^}AlM]_2_ (M = K, Rb, Cs; SiN^Dipp^ = {CH_2_SiMe_2_NDipp}_2_), display a variable kinetic facility (K < Rb < Cs) toward oxidative addition of the acidic C–H bond of terminal alkynes to provide the corresponding alkali metal hydrido(alkynyl)aluminate derivatives. Theoretical analysis of the formation of these compounds through density functional theory (DFT) calculations implies that the experimentally observed changes in reaction rate are a consequence of the variable stability of the [{SiN^Dipp^}AlM]_2_ dimers, the integrity of which reflects the ability of M^+^ to maintain the polyhapto group 1-arene interactions necessary for dimer propagation. These observations highlight that such “on-dimer” reactivity takes place sequentially and also that the ability of each constituent Al(I) center to effect the activation of the organic substrate is kinetically differentiated.

## Introduction

**Scheme 1:**
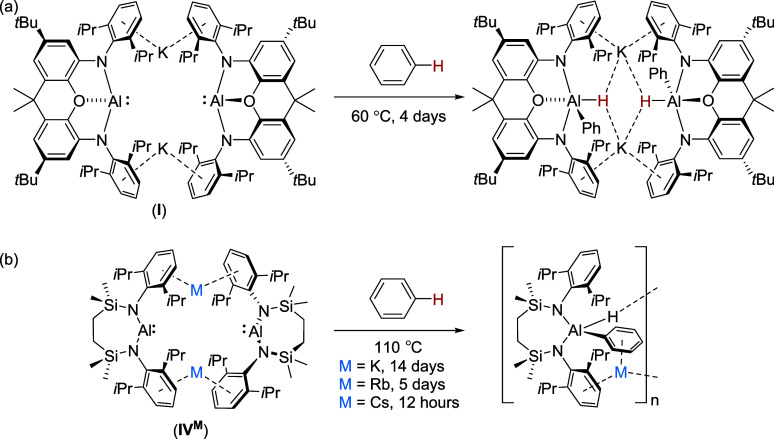
Reactivity of (a) Compound **I** and (b) Compounds **IV**^**M**^ toward the C–H Bonds of Benzene

The synthesis and reactivity of alumanyl anions has emerged as a significant topic of study during the last half decade.^1−4^ In common with many species comprising a low oxidation state *p*-block element,^5^ it was quickly recognized that the frontier orbitals presented by the Al(I) center of Aldridge, Goicoechea, and coworkers’ initially reported [K{Al(_xanth_NON)]_2_ (**I**, _xanth_NON = [4,5-(NDipp)_2_-2,7-*t*-Bu_2_-9,9-Me_2_-xanthene]^2–^, where Dipp = 2,6-*i*-Pr_2_C_6_H_3_) lend themselves to oxidative addition of even the robust C–H bonds of benzene (Scheme 1a).^6^ While the demonstrated ability of **I**, and the now broad palette of related compounds, to effect similar reductive activation of a wide variety of small molecules has continued to expand,^7−22^ the relative importance of the constituent alkali metal cation (M^+^) has received only limited consideration.^14,23,24^

Reminiscent of the reactivity depicted in Scheme 1, Coles and Mulvey initially observed that among various derivatives of the form, [(NON)AlM]_2_ (**II**^**M**^; NON = O(SiMe_2_NDipp)_2_; M = Li,^14^ Na,^14^ K,^11^ Rb,^25^ Cs^25^), only **II**^**Cs**^ induced benzene activation, providing [Cs{Al(H)(C_6_H_5_)(NON)}] after 5 days heating at 80 °C.^25a^ Coles, Fulton, and coworkers have very recently reported that the application of more forcing conditions to the same system when M = Na or K (11 and 14 days at 100 °C, respectively) does in fact induce 1,4-phenylene di(hydrido)aluminate formation by the consecutive activation of two *para*-oriented C–H bonds benzene C–H bonds.^25b^ That this latter reactivity is effected by the lighter group 1 derivatives presents a significant contrast with Harder and coworkers’ earlier report that, among their complete series of dimeric group 1 alumanyls, [{(BDI^Dipp^-H)Al}M]_2_ (**III**^**M**^; BDI^Dipp^-H = [DippNC(Me)=C(H)C(=CH_2_)(NDipp)]^2–^; M = Li, Na, K, Rb, Cs],^26^ only the three heaviest group 1 congeners, **III**^**K,Rb,Cs**^, were able to induce similar 2-fold C–H addition at benzene. Irrespective of this discrepancy, it is notable that the preferred computed mechanism of benzene activation for **II**^**Na**^ and **II**^**K**^ did not require the monomerization of the initial M···arene-bridged dimeric structures.^25b^

Parallel advances in heterobimetallic alkali metal mediation (AMM) reactivity have revealed notable gradations of metaling efficiency, particularly of arenes, that are dependent on the identity of the alkali metal.^27^ In a similar manner, the dimeric group 1 alumanyl derivatives, [{SiN^Dipp^}AlM]_2_ (**IV**^**M**^; SiN^Dipp^ = {CH_2_SiMe_2_NDipp}_2_; M = K,^28,29^ Rb,^23^ Cs^23^), also display a variable efficacy toward C–H oxidative addition of arene C–H bonds at elevated temperature (Cs > Rb > K, 110 °C) (Scheme 1b).^23^ Computational assessment of this reactivity deduced that, in common with Coles, Fulton and coworkers’ analysis of **II**^**Na**^ and **II**^**K**^, the integrity of the dimeric system is maintained throughout a mechanism in which rate determining attack of the Al(I) nucleophile is modulated by an increasing preference for M^+^ π-engagement with the arene solvent as group 1 is descended.

In a superficially similar manner, the C–H bonds of terminal alkynes have been reasoned to undergo a lowering of their p*K*_a_ by 9.8 pH units via the interaction of their polarizable π-system with Cu(I) or Au(I) cations.^30,31^ Al-centered oxidative addition to alumanyl anions has also been demonstrated for a variety of both acidic (E = N, O, P) and hydridic (E = SiH) E–H bonds.^13,21,32,33^ Although the reactivity of C–H acidic terminal alkynes with group 1 alumanyls appears to have thus far escaped attention, in related work we have observed that treatment of [{SiN^Dipp^}Al–Cu(NHC*^i^*^Pr^)] (NHC*^i^*^Pr^ = *N*,*N*′-di-isopropyl-4,5-dimethyl-2-ylidene) results in formal proton reduction and the formation of cuprous (hydrido)(alkynyl)aluminate derivatives.^34^ Reasoning that similar chemistry utilizing group 1 alumanyls may provide a further system with which to assess the impact of group 1 cation identity, we herein present a study of the C–H oxidative addition of terminal alkynes toward **IV**^**M**^ (M = K, Rb, Cs).

## Experimental Section

### General Considerations

Except stated otherwise, all the experiments were conducted using standard Schlenk line and/or glovebox techniques under an inert atmosphere of argon. NMR spectra were recorded with a Bruker Avance III spectrometer (^1^H at 400 MHz, ^13^C at 101 MHz) or an Agilent ProPulse spectrometer (^1^H at 500 MHz, ^13^C at 126 MHz). The spectra are referenced relative to residual protio solvent resonances. Elemental analyses were performed at Elemental Microanalysis Ltd., Okehampton, Devon, UK. Solvents were dried by passage through a commercially available solvent purification system and stored under argon in ampules over 4 Å molecular sieves. Benzene-*d*_6_ and THF-*d*_8_ were purchased from Sigma-Aldrich, dried over a potassium mirror before distillation and storage over molecular sieves. [{SiN^Dipp^}AlK]_2_ (**IV**^**K**^), [{SiN^Dipp^}AlRb]_2_ (**IV**^**Rb**^), and [{SiN^Dipp^}ACs]_2_ (**IV**^**Cs**^) were prepared according to reported procedures.^23,28^ All the terminal acetylenes were purchased from Merck and degassed by three freeze–pump–thaw cycles and stored over 4 Å molecular sieves for more than 18 h prior to usage. Other chemicals were purchased from Merck and used without further purification.

#### Synthesis of K_2_[{SiN^Dipp^}Al(H){C≡CC_6_H_2_(2,4,6-Me_3_)}]_2_ (**1**)

[{SiN^Dipp^}AlK]_2_ (**IV**^**K**^, 20.0 mg, 0.018 mmol) was dissolved in C_6_D_6_ (*ca*. 0.5 mL). 2-Ethynyl-1,3,5-trimethylbenzene (5.2 mg, 5.6 μL, 0.036 mmol) was added to the bright yellow benzene solution of **IV**^**K**^, and the reaction mixture was kept at 40 °C for 3 days. After removal of all volatiles, storage of a toluene/hexane (1:10) solution of the crude product at −30 °C yielded a crop of colorless block crystals of **1**. Yield 13.1 mg, 51%. Anal. Calcd For C_41_H_63_AlKN_2_Si_2_, C: 69.73%, H: 8.99%, N: 3.97%. Found: 68.63%, H: 8.68%, N: 3.87%. ^1^H NMR (500 MHz, 298 K, C_6_D_6_) δ 6.98–6.94 (m, 2H, Ar-*H*), 6.84–6.82 (m, 4H, Ar*-H*), 6.65–6.62 (m, 2H, Ar*-H*), 4.34 (sept, ^3^*J*_HH_ = 6.8 Hz, 2H, C*H*Me_2_), 4.23 (sept, ^3^*J*_HH_ = 6.5 Hz, 1H, C*H*Me_2_), 4.04–3.98 (m, 1H, C*H*Me_2_), 2.13 (s, 3H, Mes C*H*_3_), 2.04 (s, 3H, Mes C*H*_3_), 2.00 (m, 3H, Mes C*H*_3_), 1.37–1.33 (m, 12H, CH*Me*_*2*_), 1.30–1.27 (m, 12H, CH*Me*_*2*_), 0.80 (s, br, 4H, SiC*H*_*2*_) 0.37 (s, 6H, Si*Me*_*2*_), 0.37 (s, 6H, Si*Me*_*2*_) ppm. ^1^H resonance correlated to Al*H* was not observed. ^13^C{^1^H} NMR (126 MHz, 298 K, C_6_D_6_) δ 150.1 (*i-*C_6_H_3_), 149.1, 148.5 (*o-*C_6_H_3_), 145.9 (*i-*C_6_H_2_), 139.3 (*o-*C_6_H_2_), 136.4 (*p-*C_6_H_2_), 129.6 (*m-*C_6_H_2_), 123.5 (*p-*C_6_H_3_), 122.6, 122.1 (*m-*C_6_H_3_), 27.8, 27.6 (*C*HMe_2_), 27.3, 26.3, 25.6, 25.3 (CH*Me*_2_), 21.9 (*o-Me* C_6_H_2_), 21.2 (*p-Me* C_6_H_2_), 15.1 (Si*C*H_2_), 1.6, 1.3 (Si*Me*_2_) ppm. ^13^C resonances correlated to Al*CC* were not observed.

#### Synthesis of K[{SiN^Dipp^}Al(H)(C≡CSiMe_3_)] (**2**)

[{SiN^Dipp^}AlK]_2_ (**IV**^**K**^, 28.0 mg, 0.025 mmol) was dissolved in C_6_D_6_ (*ca*. 0.5 mL). Ethynyltrimethylsilane (4.9 mg, 7.1 μL, 0.05 mmol) was added to the bright yellow benzene solution of **IV**^**K**^. The reaction mixture was then kept at 40 °C for 3 days, and a colorless solution was observed as the crude reaction mixture. Gradual cooling of the solution to the room temperature induced the deposition of compound **2** as colorless crystals suitable for X-ray single crystal diffraction. Yield 25.1 mg, 76%. Anal. Calcd For C_35_H_60_AlKN_2_Si_3_, C: 63.77%, H: 9.17%, N: 4.25%. Found, C: 63.32%, H: 9.43%, N: 3.98%. ^1^H NMR (400 MHz, 298 K, *d*_8_-THF) δ 6.95 (dd, ^3^*J*_HH_ = 7.6, ^4^*J*_HH_ = 1.8 Hz, 2H, *m*-C_6_*H*_3_), 6.91 (dd, ^3^*J*_HH_ = 7.6, ^4^*J*_HH_ = 1.8 Hz, 2H, *m*-C_6_*H*_3_), 6.80 (t, ^3^*J*_HH_ = 7.6 Hz, 2H, *p*-C_6_*H*_3_), 4.15 (sept, ^3^*J*_HH_ = 6.8 Hz, 2H, C*H*Me_2_), 4.02 (sept, ^3^*J*_HH_ = 6.8 Hz, 2H, C*H*Me_2_), 1.33 (d, ^3^*J*_HH_ = 6.8 Hz, 6H, CH*Me*_2_), 1.22 (d, ^3^*J*_HH_ = 6.8 Hz, 6H, CH*Me*_2_), 1.15* (d, ^3^*J*_HH_ = 6.8 Hz, 6H, CH*Me*_2_), 1.14* (d, ^3^*J*_HH_ = 6.8 Hz, 6H, CH*Me*_2_) *overlapping peaks, 0.92 (s, 4H, SiC*H*_2_), −0.02 (s, 6H, Si*Me*_2_), −0.03 (s, 6H, Si*Me*_2_), −0.26 (s, 9H, Si*Me*_3_) ppm. ^1^H resonance correlated to Al*H* was not observed. ^13^C{^1^H} NMR (101 MHz, 298 K, *d*_8_-THF) δ 150.3 (*i*-*C*_6_H_3_), 148.5 (*o*-*C*_6_H_3_), 148.5 (*o*-*C*_6_H_3_), 123.6 (*m*-*C*_6_H_3_), 123.5 (*m*-*C*_6_H_3_), 121.8 (*p*-*C*_6_H_3_), 28.1 (*C*HMe_2_), 27.8 (*C*HMe_2_), 26.7 (CH*Me*_2_), 26.7 (CH*Me*_2_), 26.2 (CH*Me*_2_), 15.9 (Si*C*H_2_), 1.3 (Si*Me*_2_), 1.2 (Si*Me*_2_), 0.9 (Si*Me*_3_) ppm. ^13^C resonances correlated to Al*CC* were not observed.

#### Synthesis of K[{SiN^Dipp^}Al(H)(C≡C*^t^*Bu)] (**3**)

[{SiN^Dipp^}AlK]_2_ (**IV**^**K**^, 50.0 mg, 0.045 mmol) was dissolved in C_6_D_6_ (*ca*. 0.5 mL). 3,3-Dimethyl-1-butyne (7.3 mg, 11.0 μL, 0.089 mmol) was added to the bright yellow *d*_6_-benzene solution of **IV**^**K**^. The reaction mixture was then kept at 40 °C for 3 days, and a colorless solution was observed as the crude reaction mixture. Slow evaporation of the solution at the room temperature allows the deposition of a crop of colorless block crystals as the product **3**. Yield 40.2 mg, 68%. A single crystal suitable for X-ray diffraction analysis could be selected from the deposited crystalline solids. Anal. Calcd For C_36_H_60_AlKN_2_Si_2_, C: 67.23%, H: 9.40%, N: 4.36%. Found, C: 67.37%, H: 9.35%, N: 4.19%. ^1^H NMR (500 MHz, 298 K, *d*_8_-THF) δ 6.96 (dd, ^3^*J*_HH_ = 7.5, ^4^*J*_HH_ = 1.8 Hz, 2H, *m*-C_6_*H*_3_), 6.92 (dd, ^3^*J*_HH_ = 7.5, ^4^*J*_HH_ = 1.8 Hz, 2H, *m*-C_6_*H*_3_), 6.80 (t, ^3^*J*_HH_ = 7.5 Hz, 2H, *p*-C_6_*H*_3_), 4.14 (sept, ^3^*J*_HH_ = 6.9 Hz, 2H, C*H*Me_2_), 4.03 (sept, ^3^*J*_HH_ = 6.9 Hz, 2H, C*H*Me_2_), 1.35 (d, ^3^*J*_HH_ = 6.9 Hz, 6H, CH*Me*_*2*_), 1.24 (d, ^3^*J*_HH_ = 6.9 Hz, 6H, CH*Me*_*2*_), 1.16 (d, ^3^*J*_HH_ = 6.9 Hz, 6H, CH*Me*_*2*_), 1.14 (d, ^3^*J*_HH_ = 6.9 Hz, 6H, CH*Me*_*2*_), 0.92 (s, 4H, SiC*H*_*2*_), 0.70 (s, 9H, C(C*H*_3_)_3_), −0.02 (s, 6H, Si*Me*_*2*_), −0.03 (s, 6H, Si*Me*_*2*_) ppm. ^1^H resonance correlated to Al*H* was not observed. ^13^C{^1^H} NMR (126 MHz, 298 K, *d*_8_-THF) δ 150.7 (*i-*C_6_H_3_), 148.7, 148.6 (*o-*C_6_H_3_), 123.6, 123.5 (*m-*C_6_H_3_), 121.7 (*p-*C_6_H_3_), 31.9 (C(*C*H_3_)_3_), 28.2, 27.8 (*C*HMe_2_), 26.8, 26.6 26.1, 25.6 (CH*Me*_2_), 16.0 (Si*C*H_2_), 1.2, 1.2 (Si*Me*_2_) ppm. Quaternary carbons were not observed.

#### Synthesis of Rb[{SiN^Dipp^}Al(H)(C≡CSiMe_3_)] (**4**)

[{SiN^Dipp^}AlRb]_2_ (**IV**^**Rb**^, 30.3 mg, 0.025 mmol) was dissolved in C_6_D_6_ (*ca*. 0.5 mL). Ethynyltrimethylsilane (4.9 mg, 7.1 μL, 0.05 mmol) was added to the bright yellow benzene solution of **IV**^**Rb**^. The reaction mixture was then kept at ambient temperature overnight, and a colorless solution was observed to form. Gradual cooling of the solution to room temperature induced the deposition of compound **4** as colorless crystals suitable for X-ray single crystal diffraction analysis. Yield 24.8 mg, 70%. Anal. Calcd For C_35_H_60_AlRbN_2_Si_3_, C: 59.58%, H: 8.57%, N: 3.97%. Found, C: 59.18%, H: 8.30%, N: 4.03%. ^1^H NMR (400 MHz, 298 K, *d*_8_-THF) δ 6.96 (dd, ^3^*J*_HH_ = 7.4, ^4^*J*_HH_ = 1.9 Hz, 2H, *m*-C_6_*H*_3_) d, *J* = 7.4, 1.9 Hz, 1H), 6.95–6.91 (dd, ^3^*J*_HH_ = 7.4, ^4^*J*_HH_ = 1.9 Hz, 2H, *m*-C_6_*H*_3_), 6.82 (t, ^3^*J*_HH_ = 7.4 Hz, 2H, *p*-C_6_*H*_3_), 4.13 (sept, ^3^*J*_HH_ = 6.7 Hz, 2H, C*H*Me_2_), 4.01 (sept, ^3^*J*_HH_ = 6.7 Hz, 2H, CH*Me*_2_), 1.34 (d, ^3^*J*_HH_ = 6.7 Hz, 6H, CH*Me*_2_), 1.23 (d, ^3^*J*_HH_ = 6.7 Hz, 6H, CH*Me*_2_), 1.16* (d, ^3^*J*_HH_ = 6.7 Hz, 6H, CH*Me*_2_), 1.15* (d, ^3^*J*_HH_ = 6.7 Hz, 6H, CH*Me*_2_) *overlapping peaks, 0.93 (s, 4H, SiC*H*_2_), 0.01 (s br, 12H, Si*Me*_2_), −0.27 (s, 9H, Si*Me*_3_) ppm. ^1^H resonance correlated to Al*H* was not observed. ^13^C{^1^H} NMR (101 MHz, 298 K, *d*_8_-THF) δ 150.2 (*i-C*_6_H_3_), 148.6 (*o-C*_6_H_3_), 148.5 (*o-C*_6_H_3_), 123.6 (*m-C*_6_H_3_), 123.5 (*m-C*_6_H_3_), 121.8 (*p-C*_6_H_3_), 28.1 (*C*HMe_2_), 27.7 (*C*HMe_2_), 26.5 (CH*Me*_2_), 26.3 (CH*Me*_2_), 26.1 (CH*Me*_2_), 15.8 (Si*C*H_2_), 1.0 (Si*Me*_2_), 0.6 (Si*Me*_3_) ppm. ^13^C resonances correlated to Al*CC* were not observed.

#### Synthesis of Rb[{SiN^Dipp^}Al(H)(C≡C*^t^*Bu)] (**5**)

[{SiN^Dipp^}AlRb]_2_ (**IV**^**Rb**^, 30.3 mg, 0.025 mmol) was dissolved in C_6_D_6_ (*ca*. 0.5 mL), 3,3-Dimethyl-1-butyne (4.1 mg, 6.1 μL, 0.05 mmol) was added to the bright yellow *d*_6_-benzene solution of **IV**^**Rb**^ via a micropipette. The reaction mixture was then kept at ambient temperature, and a colorless solution was observed after 18 h. Removal of all volatiles *in vacuo* and redissolution of the residual white powder in a toluene/hexane (1:10) solvent provided a clear colorless solution, which yields compound **5** as colorless crystals when kept at −35 °C for 3 days. A single crystal suitable for X-ray diffraction analysis was selected from the obtained crystalline solids. Yield 27.3 mg, 79%. Anal. Calcd For C_36_H_60_AlRbN_2_Si_2_, C: 62.71%, H: 8.77%, N: 4.06%. Found, C: 61.77%, H: 8.51%, N: 4.39%. ^1^H NMR (400 MHz, 298 K, *d*_8_-THF) δ 6.97 (dd, ^3^*J*_HH_ = 7.5, ^4^*J*_HH_ = 1.8 Hz, 2H, *m*-C_6_*H*_3_), 6.93 (dd, ^3^*J*_HH_ = 7.5, ^4^*J*_HH_ = 1.8 Hz, 2H, *m*-C_6_*H*_3_), 6.82 (t, ^3^*J*_HH_ = 7.6 Hz, 2H, *p*-C_6_*H*_3_), 4.15 (sept, ^3^*J*_HH_ = 6.8 Hz, 2H, C*H*Me_2_), 4.02 (sept, ^3^*J*_HH_ = 6.8 Hz, 2H, C*H*Me_2_), 1.35 (d, ^3^*J*_HH_ = 6.8 Hz, 6H, CH*Me*_2_), 1.25 (d, ^3^*J*_HH_ = 6.8 Hz, 6H, CH*Me*_2_), 1.16* (d, ^3^*J*_HH_ = 6.8 Hz, 6H, CH*Me*_2_) 1.15* (d, ^3^*J*_HH_ = 6.8 Hz, 6H, CH*Me*_2_) *overlapping peaks, 0.92 (s, 4H, SiC*H*_2_), 0.70 (s, 9H, C*Me*_3_), −0.02 (s, 6H, Si*Me*_2_), −0.03 (s, 6H, Si*Me*_2_) ppm. ^1^H resonance correlated to Al*H* was not observed. ^13^C{^1^H} NMR (101 MHz, 298 K, *d*_8_-THF) δ 150.8 (*i-C*_6_H_3_), 148.8 (*o-C*_6_H_3_), 148.7 (*o-C*_6_H_3_), 123.8 (*m-C*_6_H_3_), 123.5 (*m-C*_6_H_3_), 121.8 (*p-C*_6_H_3_), 31.8 (C*Me*_3_), 28.2 (*C*HMe_2_), 27.8 (*C*HMe_2_), 26.7 (CH*Me*_2_), 26.5 (CH*Me*_2_), 16.0 (Si*C*H_2_), 1.2 (Si*Me*_2_), 1.1 (Si*Me*_2_) ppm. Quaternary carbons were not observed.

#### Synthesis of Cs[{SiN^Dipp^}Al(H)(C≡CSiMe_3_)] **(6**)

[{SiN^Dipp^}AlCs]_2_ (**IV**^**Cs**^, 16.4 mg, 0.013 mmol) was dissolved in C_6_D_6_ (*ca*. 0.5 mL). Ethynyltrimethylsilane (2.5 mg, 3.5 μL, 0.026 mmol) was then added to the bright yellow benzene solution of **IV**^**Cs**^ via a micropipette. The reaction mixture was kept at ambient temperature, and a colorless solution was obtained within 1 h. Slow evaporation of the benzene solution provides compound **6** as colorless crystalline needles suitable for X-ray diffraction analysis. Yield 10.2 mg, 54%. No meaningful result for elemental analysis was obtained after several attempts. ^1^H NMR (500 MHz, 298 K, Benzene-*d*_6_) δ 7.06–6.94 (m, 4H, *m*-C_6_*H*_3_), 6.89–6.79 (m, 2H, *p*-C_6_*H*_3_), 4.43–4.21 (m, 2H, C*H*Me_2_), 4.21–4.00 (m, 2H, C*H*Me_2_), 1.39 (d_app_, *J* = 6.8 Hz, 12H, CH*Me*_2_), 1.25 (dd, *J* = 6.8 Hz, 12H, CH*Me*_2_), 1.15–0.99 (m, 4H, SiC*H*_2_), 0.39 (s br, 6H, Si*Me*_2_), 0.29 (s br, 6H, Si*Me*_2_), 0.01 (s br, 9H, Si*Me*_3_). ^1^H resonance correlated to Al*H* was not observed. ^13^C{^1^H} NMR (126 MHz, 298 K, Benzene-*d*_6_) δ 149.5 (Ar*C*), 148.8 (Ar*C*), 148.4 (Ar*C*), 123.3 (Ar*C*), 123.1 (Ar*C*), 121.7 (Ar*C*), 31.6 (*C*HMe_2_), 27.0 (*C*HMe_2_), 25.7 (CH*Me*_2_), 25.3 (CH*Me*_2_), 25.0 (CH*Me*_2_), 22.6 (CH*Me*_2_), 13.9 (Si*C*H_2_), 1.0 (Si*Me*_2_), 0.7 (Si*Me*_2_), 0.3 (Si*Me*_3_). 13C resonances correlated to Al*CC* were not observed.

#### Synthesis of Cs[{SiN^Dipp^}Al(H)(C≡C*^t^*Bu)] (**7**)

[{SiN^Dipp^}AlCs]_2_ (**IV**^**Cs**^, 16.4 mg, 0.013 mmol) was dissolved in C_6_D_6_ (*ca*. 0.5 mL). 3,3-Dimethyl-1-butyne (2.1 mg, 3.1 μL, 0.026 mmol) was added to the bright yellow *d*_6_-benzene solution via a micropipette. The reaction mixture was then kept at ambient temperature, and a colorless solution was obtained within 1 h. Slow evaporation of the benzene solution provides compound **7** as colorless crystals suitable for X-ray diffraction analysis. Yield 11.5 mg, 62%. Anal. Calcd For C_36_H_60_AlCsN_2_Si_2_, C: 58.67%, H: 8.21%, N: 3.80%. Found, C: 57.77%, H: 7.88%, N: 3.80%. ^1^H NMR (400 MHz, THF-*d*_8_) δ 6.98–6.90 (m, 4H, *m*-C_6_*H*_3_), 6.81 (t, ^3^*J*_HH_ = 7.5 Hz, 2H, *p*-C_6_*H*_3_), 4.14 (sept, ^3^*J*_HH_ = 6.9 Hz, 2H, C*H*Me_2_), 4.05 (sept, ^3^*J*_HH_ = 6.9 Hz, 2H, C*H*Me_2_), 1.35 (d, ^3^*J*_HH_ = 6.9 Hz, 6H, CH*Me*_2_), 1.26 (d, ^3^*J*_HH_ = 6.9 Hz, 6H, CH*Me*_2_), 1.16* (d, ^3^*J*_HH_ = 6.9 Hz, 6H, CH*Me*_2_), 1.15* (d, ^3^*J*_HH_ = 6.9 Hz, 6H, CH*Me*_2_) *overlapping peaks, 0.92 (s, 4H, SiC*H*_2_), 0.70 (s, 9H, C*Me*_3_), −0.02 (s, 6H, Si*Me*_2_), −0.03 (s, 6H, 6H, Si*Me*_2_) ppm. ^1^H resonance correlated to Al*H* was not observed. ^13^C{^1^H} NMR (101 MHz, 298 K, *d*_8_-THF) δ 151.0 (*i-C*_6_H_3_), 148.9 (*o-C*_6_H_3_), 148.7 (*o-C*_6_H_3_), 123.8 (*m-C*_6_H_3_), 123.5 (*m-C*_6_H_3_), 121.8 (*p-C*_6_H_3_), 31.6 (C*Me*_3_), 28.4 (*C*Me_3_), 28.2 (*C*HMe_2_), 27.7 (*C*HMe_2_), 26.4 (CH*Me*_2_), 26.4 (CH*Me*_2_), 15.9 (Si*C*H_2_), 1.0 (Si*Me*_2_), 0.9 (Si*Me*_2_) ppm. ^13^C resonances correlated to Al*CC* were not observed.

## Results and Discussion

### Synthesis

**Scheme 2:**
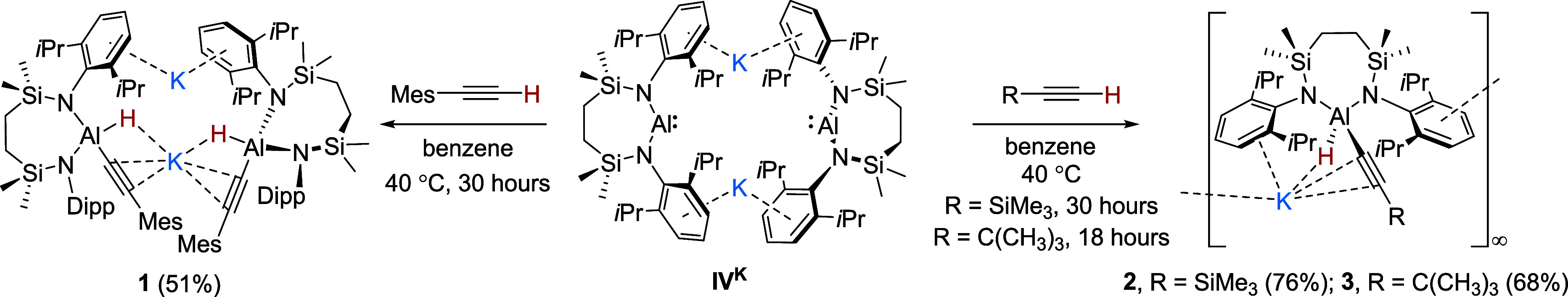
Synthesis of Compounds **1**–**3** with Isolated % Yields Indicated in Parentheses

**Figure 1:**
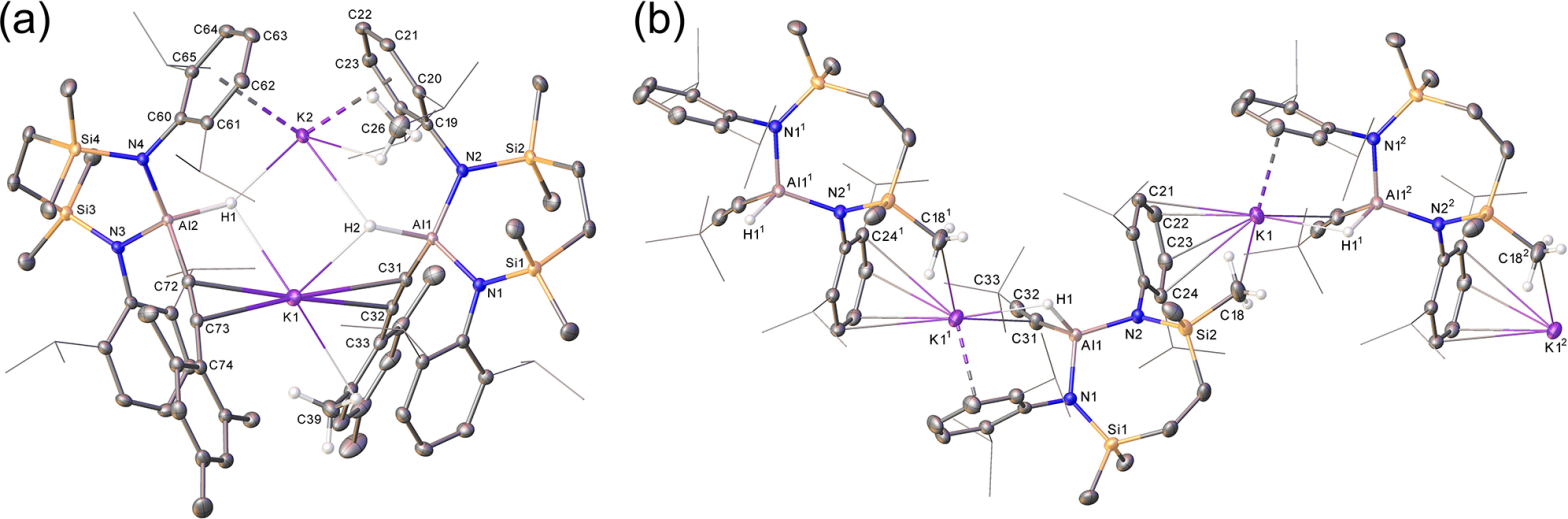
Molecular structures of (a) compound **1** and (b) compound **3** with displacement ellipsoids at 30%. For clarity, hydrogen atoms, aside from those bonded to aluminum, and occluded toluene solvent (**1**) are omitted and selected aryl and alkyl substituents are displayed as wireframe. Symmetry operations to generate primed atoms for **3**: ^1^1/2 – *x*, 1/2 + *y*, +*z*; ^2^1/2 – *x*, −1/2 + *y*, +*z*.

Initial synthetic experiments assayed the reactivity of the potassium alumanyl [{SiN^Dipp^}AlK]_2_ (**IV**^**K**^) with three terminal alkynes, RC≡CH (R = 2,4,6-Me_3_C_6_H_2_; Me_3_Si; (CH_3_)_3_C), at 40 °C (Scheme 2). Monitoring of the progress of each reaction by ^1^H NMR spectroscopy indicated that, while stoichiometric conversion to the respective mesityl- and trimethylsilyl-substituted derivatives, **1** and **2**, required 30 h, the reaction with 3,3-dimethyl-1-butyne to provide **3** was somewhat more facile, reaching completion within 18 h. Although the resultant Al–H resonances could not be identified during the solution-state analysis, all three compounds were confirmed as the anticipated potassium hydrido(alkynyl)aluminates by single crystal X-ray diffraction. The resultant structures of **1** and **3** are presented in Figure 1, while that of **2** is depicted in Figure S24. Selected bond length and angle data for all three compounds are provided in Table 1.

**Table 1:** Selected Bond Lengths (Å) and Angles (deg) for Compounds **1**–**3**

	**1**	**2**	**3**
Al1–N1	1.8639(13)	1.870(2)	1.8705(18)
Al1–N2	1.8597(13)	1.862(2)	1.8652(19)
Al1–C31	1.9821(16)	1.977(3)	1.980(3)
K1–C31	3.0232(15)	3.273(3)^a^	3.260(3)^g^
K1–C32	3.2252(15)		
C31–C32	1.215(2)	1.224(5)	1.196(4)
Al2–N3	1.8661(12)	1.874(2)	
Al2–N4	1.8579(13)	1.858(2)	
Al2–C72	1.9839(16)	1.976(3)^b^	
K1–C72	2.9969(15)	3.217(3)^c^	
K1–C73	3.2871(15)		
C72–C73	1.213(2)	1.211(5)^d^	
N1–Al1–N2	113.48(6)	114.51(10)	113.89(8)
N1–Al1–C31	106.89(6)	113.30(11)	112.31(10)
N2–Al1–C31	114.73(6)	106.62(11)	107.50(9)
N3–Al2–N4	114.22(6)	114.66(11)	
N3–Al2–C72	106.84(6)	112.33(11)^e^	
N4–Al2–C72	113.71(6)	107.56(12)^f^	

a K1–C66.

b Al2–C66.

c K2–C31^1^.

d C66–C67.

e N3–Al2–C66.

f N4–Al2–C66.

g K1–C31^1^.

Compound **1** crystallizes from toluene as a noncentrosymmetric dimer of [{SiN^Dipp^}Al(H)C≡C(2,4,6-Me_3_C_6_H_2_)]^−^ anions connected by two potassium cations. Although both cations display close contacts to the hydridic hydrogens, K1 interacts primarily via close but asymmetric η^2^-engagement with the Al1- and Al2-bound acetylide substituents, whereas K2 is encapsulated by η^6^-π-arene interactions with both Dipp substituents. In contrast to this molecular dimer, the solid-state structures of both compounds **2** and **3** present as infinite 1-dimensional polymers. Although not isostructural, the individual aluminate anions of both **2** and **3** engage with each potassium through the aluminum-bound hydride, the acetylide C≡C π bond and one Dipp substituent of the {SiN^Dipp^} spectator anion. Polymer propagation is then achieved through an additional intermolecular polyhapto interaction with a further Dipp substituent and close C–H contacts to a silylmethyl group of an adjacent aluminate. While we attribute no great significance to these solid-state variations beyond the relative kinetic impact of the alkyne *C*-substituent steric demands, it is notable that the resultant close contacts between each potassium and the acetylide substituents impose a negligible impact on the lengths of the C≡C triple bonds across all three structures [**1** C31–32 1.215(2), C72–C73 1.213(2); **2** C31–32 1.224(5), C66–C67 1.211(5); **3** C31–32 1.196(4) Å]. These measurements also bear comparison to the corresponding metrics in the terminal acetylide components of the charge neutral compounds, [(BDI^Dipp^)Al(H)C≡CPh] (BDI^Dipp^ = HC{(Me)CNDipp}_2_; 1.203(5) Å) and [(Me_3_C)_2_Al(μ-H)[μ-C≡C-2,4,6-(Me_3_C)_3_C_6_H_2_]Al(CMe_3_)[C≡C-2,4,6-(Me_3_C)_3_C_6_H_2_] (1.213(4) Å), which provide the only previously described examples of molecular hydridoaluminum alkynides.^35,36^

**Scheme 3:**
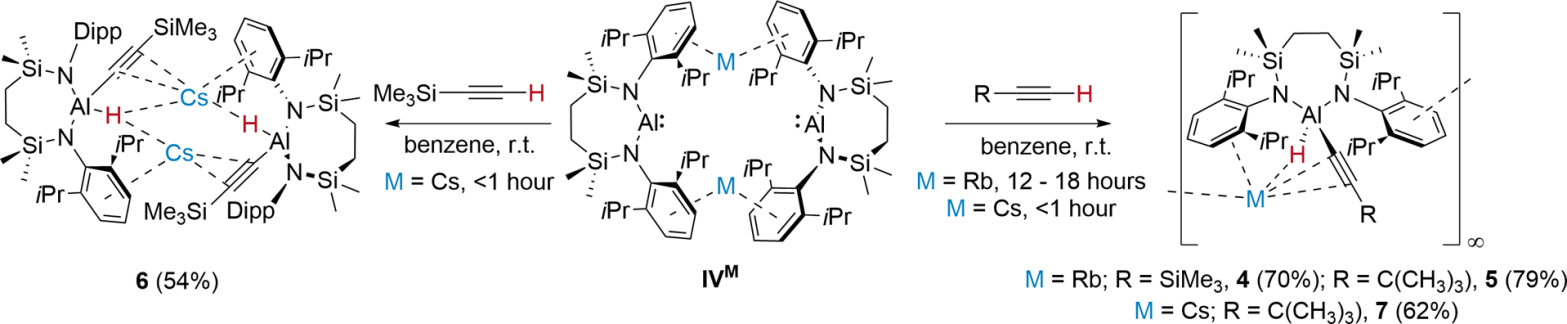
Synthesis of Compounds **4**–**7** with Isolated % Yields Indicated in Parentheses

Once crystallized, all three potassium aluminate derivatives displayed only limited solubility in noncoordinating arene solvents. Further characterization of compounds **1**–**3** was, thus, achieved by ^1^H and ^13^C NMR spectroscopy in *d*_8_-THF solution, whereupon the relatively simple and readily diagnostic spectra of compounds **2** and **3** (see Supporting Information) directed our investigation of the heavier rubidium and cesium alumanyls exclusively toward the trimethylsilyl- and *tert*-butylalkynide derivatives. Accordingly, reactions of **IV**^**Rb**^ with both ethynyltrimethylsilane and 3,3-dimethyl-1-butyne proceeded to completion to provide compounds **4** and **5** with no necessity for external heating during 12–18 h. A similar enhancement in reaction rate was apparent for analogous reactions performed with **IV**^**Cs**^, which resulted in the generation of compounds **6** and **7**, in both cases within 1 h at ambient temperature (Scheme 3).

**Figure 2:**
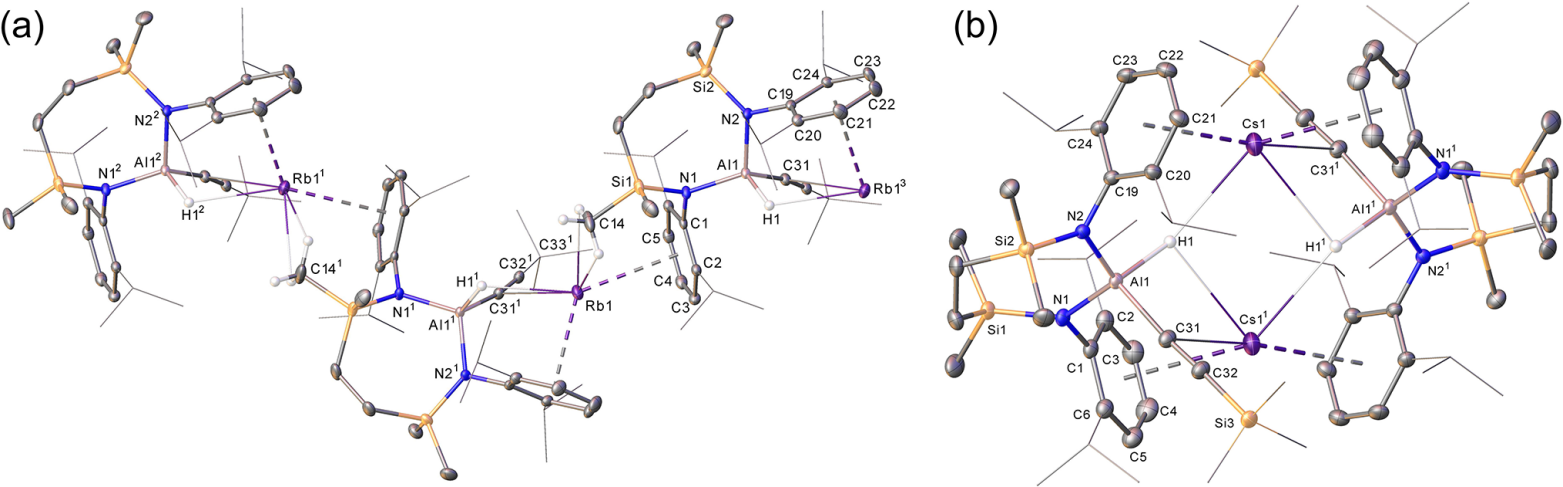
Molecular structures of (a) compound **5** and (b) compound **6** with displacement ellipsoids at 30%. For clarity, hydrogen atoms, aside from those bonded to aluminum, and disordered atoms (**6**) are omitted, and selected aryl and alkyl substituents are displayed as wireframe. Symmetry operations to generate primed atoms for **5**, ^1^3/2 – *x*, −1/2 + *y*, +*z*, and for **6**, ^1^1 – *x*, 1 – *y*, 1 – *z*.

Compounds **4**–**7** presented ^1^H and ^13^C{^1^H} NMR spectra that were indicative of similar solution structures and reminiscent of the analogous data provided by the corresponding potassium derivatives, **2** and **3**. Single crystals of all four compounds were also obtained, allowing the identification of their solid-state structures by X-ray diffraction analysis. The representative structures of the heavier alkali metal compounds, **5** and **6**, are illustrated in Figure 2 (compounds **4** and **7** are shown in Figures S25 and S26, respectively), while selected bond length and angle data for **5**–**7** are presented in Table 2. Although the data collected for **4** were of sufficient quality to only establish connectivity, this species and both **5** and **7** were again found to crystallize as 1-dimensional polymers, whereby the individual Rb and Cs hydrido(alkynyl)aluminate units assemble as infinite arrays through sequences of polyhapto contacts between the alkali metal cations and a Dipp substituent of a proximal {SiN^Dipp^} ligand. In contrast, the *C*-silylated cesium derivative (**6**) adopts a dimeric molecular structure. Although this latter motif is reminiscent of that identified for compound **1**, the structure of **6** is centrosymmetric and both heavier group 1 centers are encapsulated by an identical combination of Al–H···Cs, η^2^-C≡C···Cs and two polyhapto-Dipp interactions across both dimer halves.

**Table 2:** Selected Bond Lengths (Å) and Angles (deg) for Compounds **5**–**7**

	**5**^a^	**6**^b^	**7**^b^
Al1–N1	1.8674(16)	1.8649(17)	1.864(3)
Al1–N2	1.8690(16)	1.8720(17)	1.868(3)
Al1–C31	1.970(2)	2.003(2)	1.976(4)
M1-C31	3.300(2)^c^	3.497(5)^d^^,^^e^	3.379(4)
C31–C32	1.206(3)	1.216(3)	1.201(6)
N1–Al1–N2	113.94(7)	114.43(8)	114.68(14)
N1–Al1–C31	107.60(8)	110.21(8)	107.30(15)
N2–Al1–C31	113.02(8)	111.21(8)	112.44(15)

a M = Rb.

b M = Cs.

c Rb1–C31^1^.

d Cs1A^1^–C31.

e Cs1^1^–C31 3.564(2).

### Mechanistic Rationale

**Figure 3:**
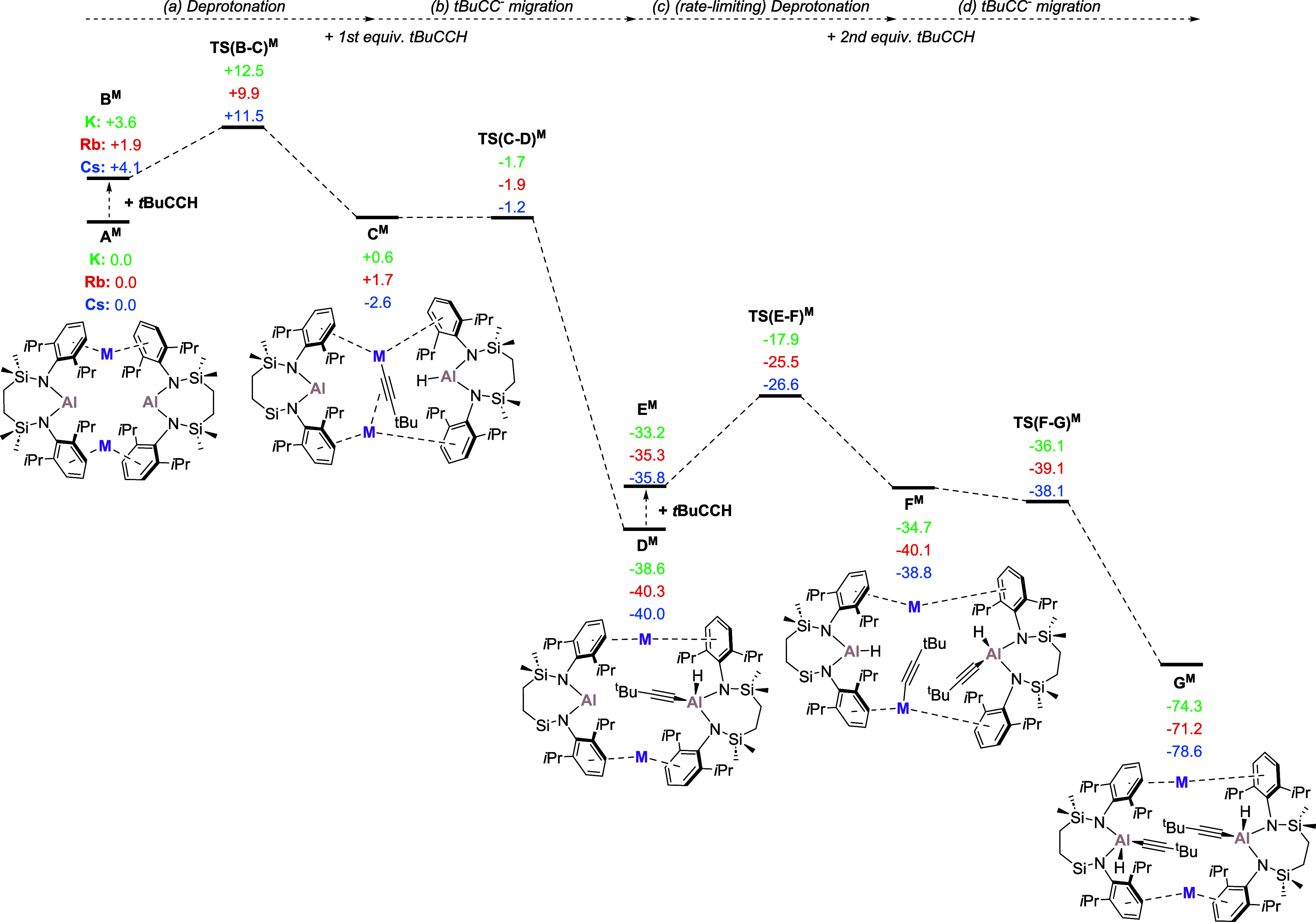
Computed free energies (kcal mol^–1^) of addition and C–H activation of *t*-BuC≡CH at various {Al_2_M_2_} species via acetylene deprotonation, where M = K (green), Rb (red), and Cs (blue), computed at the BP86-D3^BJ^(PCM = Benzene)/BS2//BP86/BS1 level of theory.^37^.

Density functional theory (DFT) calculations were performed to provide mechanistic insight into the oxidative addition of *t-*BuC≡CH, leading to the hydrido(alkynyl)aluminate derivatives, **3**, **5** and **7**. Specific scrutiny was directed to the apparent variation in kinetic aptitude of **IV**^**M**^ (henceforth, labeled “**A**^**M**^” in the computational study) to effect this transformation across the three dissimilar alkali metals (i.e., M = K, Rb, Cs). In a manner reminiscent of our previous analysis of the benzene-derived reactivity shown in Scheme 1b, the kinetically preferred pathways were characterized to proceed without any necessary dissociation from the dimeric constitution of **A**^M^ (Figure 3). Transition state geometries were, thus, identified from **A**^**M**^, where formation of an initial C–H activation product via **TS(B–C)**^**M**^ invokes direct deprotonation of the terminal acetylene by the alumanyl base. The generation of the transient alkali metal acetylide adduct (**C**^**M**^) exhibits a similar kinetic facility irrespective of the identity of M, with low energetic spans relative to the preceding reactant, **A**^**M**^, and *t-*BuC≡CH (M = K, 12.5; Rb, 9.9; and Cs 11.5 kcal mol^–1^). We suggest, therefore, that the minor variations computed across the three alkali metal systems are more reflective of the relative ease of partial disruption of the dimeric structures of **A**^**M**^ (*vide infra*) than any direct impact on the ability of the alumanyl center to effect the deprotonation. Although the transiently formed alkali metal acetylide adducts **C**^**M**^ undergo effectively barrierless onward transformation to the hemihydrido(alkynyl)aluminate species (**D**^**M**^) the presence of the remaining formal Al(I) center of **D**^**M**^ dictates that they remain liable to analogous C–H addition with a further equivalent of *t-*BuC≡CH. The energetic spans associated with the resultant deprotonation transition states [**TS(E-F)**^**M**^; M = K, 20.7; Rb, 14.8; and Cs 13.4 kcal mol^–1^], thus, identify this process as rate determining and now indicate an order of reactivity of (K < Rb < Cs) that is in qualitative agreement with experiment. In an analogous manner to the formation of **C**^**M**^, the remaining Al(I) center of the intermediate, **E**^**M**^, was computed to effect the deprotonation of a second alkyne substrate to yield **F**^**M**^. The resultant alkynyl moiety is initially accommodated at one of the M^+^ centers, prior to the barrierless isomerization of **F**^**M**^ to the ultimate hydrido(alkynyl)aluminate products (**G**^**M**^). Although a variety of factors were assessed to interrogate the impact of variation of M during the assembly of **TS(E-F)**^**M**^, no direct influence on the reactivity of the participating C–H bond, either kinetic or electronic, could be definitively identified. Rather, and consistent with the relative strengths of M^+^–arene interactions observed experimentally by Armentrout and coworkers,^38^ we suggest that reactivity across the various group 1 alumanyl complexes is again dictated by a sequential lowering of the energetic cost incurred during the conformational adjustment of the dimeric structure of **E**^**M**^ (M = K > Rb > Cs) and its consequent aptitude to accommodate the second addition of *t-*BuC≡CH. From this perspective, the observed variation in reaction rate is best ascribed to an increasing lability to deformation and access to the Al_2_M_2_ tetrametallic core that arises from a decline in the strength of the M^+^···Dipp interactions as M^+^ increases in size and atomic mass.

## Conclusions

The group 1 alumanyls, [{SiN^Dipp^}AlM]_2_ (M = K, Rb, Cs), display a variable kinetic facility (K < Rb < Cs) toward oxidative addition of the acidic C–H bond of terminal alkynes to provide the corresponding alkali metal hydrido(alkynyl)aluminate derivatives. In contrast to our, and others’, previous deductions regarding the superficially analogous activation of benzene,^8,23,25^ this modulation of reactivity could not be traced to any direct impact of M on either the substrate C≡C or C–H bonds. Rather, we deduce that the experimentally observed changes in reaction rate are a direct consequence of the robust structures of [{SiN^Dipp^}AlM]_2_, the variable integrity of which reflect the ability of M^+^ to maintain the polyhapto group 1-arene interactions necessary for dimer propagation. These observations highlight that such “on-dimer” reactivity takes place sequentially and also that the ability of each constituent Al(I) center to effect the activation of the organic substrate is kinetically differentiated. We are continuing to investigate whether these observations may be exploited to effect further novel transformations.

## Supporting Information Available

NMR spectra; details of the X-ray and computational analyses (PDF)
Coordinates for all computed structures (XYZ)

## Supplementary Material

om4c00435_si_001.pdf

om4c00435_si_002.xyz
