# Supplementary material for: Terminal Alkyne Activation by an Al(I)-Centered Anion: Impact on the Mechanism of Alkali Metal Identity
Source: Organometallics. 2024 Dec 9;44(1):236–43. doi: 10.1021/acs.organomet.4c00435 (PMC11734126; doi:10.1021/acs.organomet.4c00435)
Supplement: Supplementary file 1 — om4c00435_si_001.pdf [file om4c00435_si_001.pdf]

Supplementary Information

for

Terminal Alkyne Activation by an Al(I)-centred Anion: Impact on Mechanism of Alkali Metal Identity

Han-Ying Liu, Henry T. W. Shere, Samuel E. Neale, Michael S. Hill,* Mary F. Mahon and Claire L. M[c]Mullin*

Department of Chemistry, University of Bath, Claverton Down, Bath, BA2 7AY, UK

Email: msh27@bath.ac.uk; cm2025@bath.ac.uk

Figure S1: ^{1}H NMR (500 MHz, 298 K, Benzene-d_6) spectrum of **1**.*Toluene, # hexane, x silicone grease.

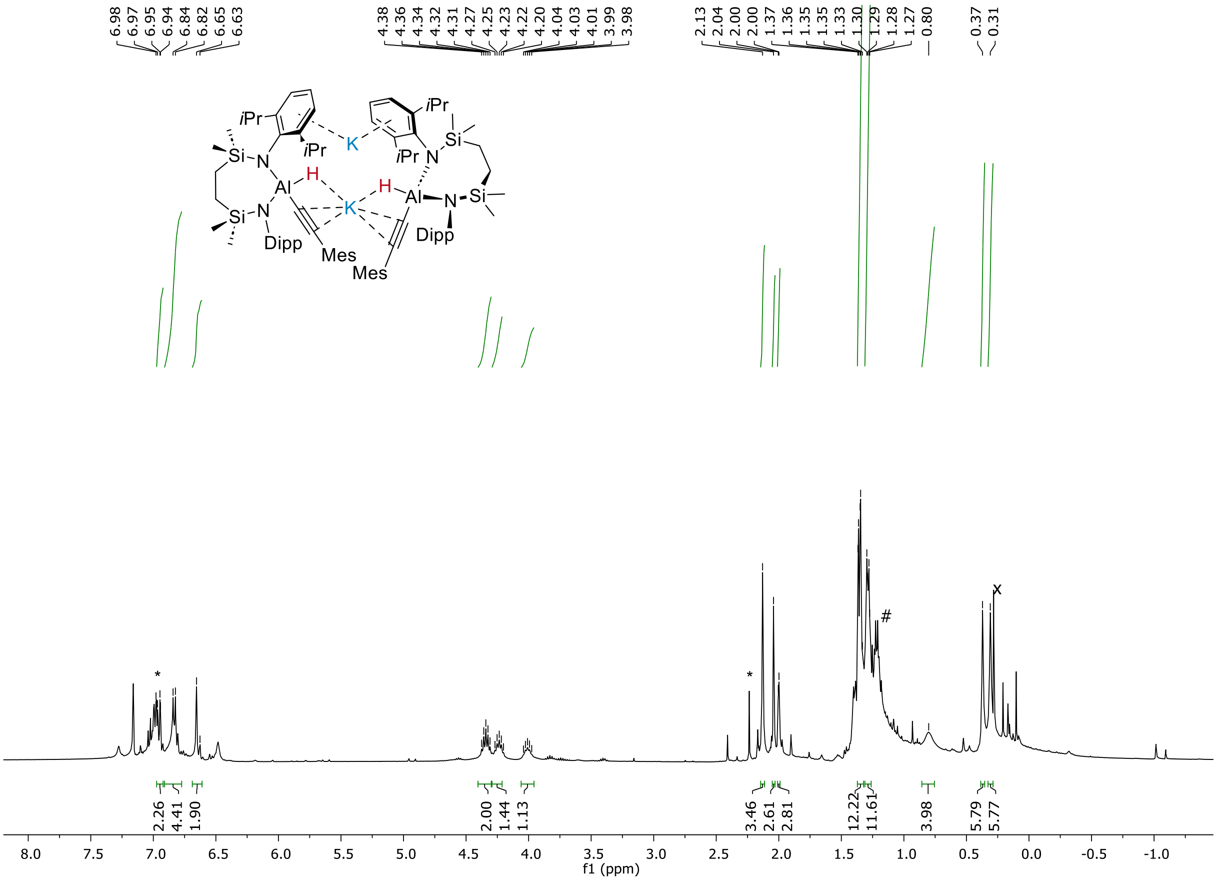

Figure S2: ^{13}C{^{1}H} NMR (126 MHz, 298 K, Benzene-d_6) spectrum of **1**.

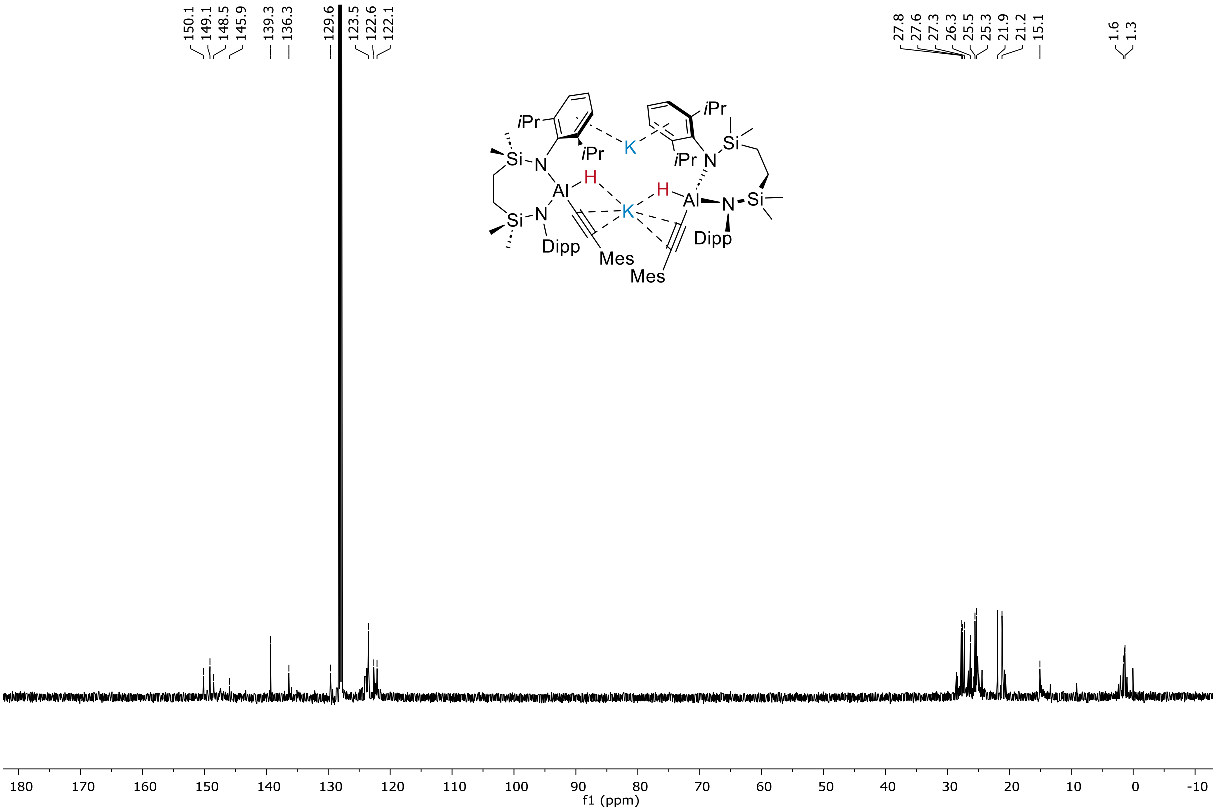

Figure S3: ^{1}H NMR (400 MHz, 298 K, d_8-THF) spectrum of **2**.

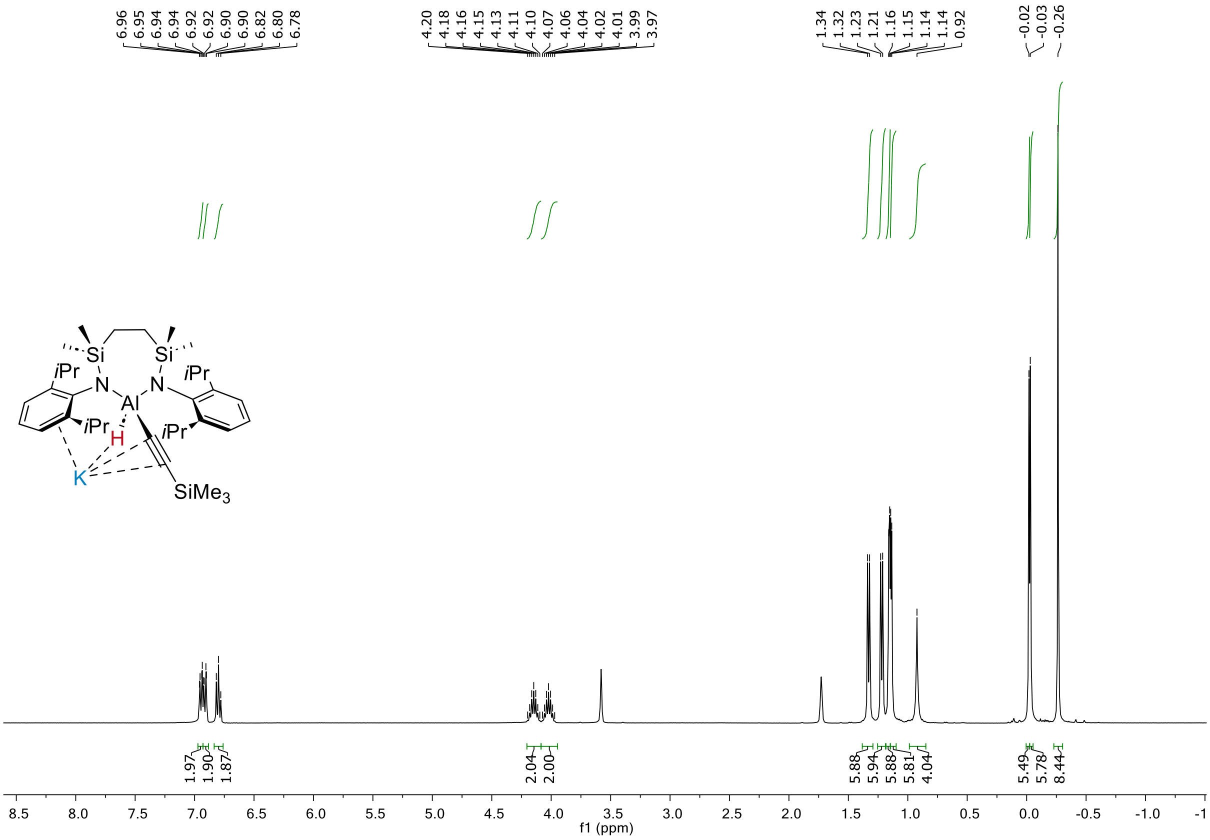

Figure S4: $^{13}C\{^{1}H\}$ NMR (101 MHz, 298 K, d_8-THF) spectrum of **2**.

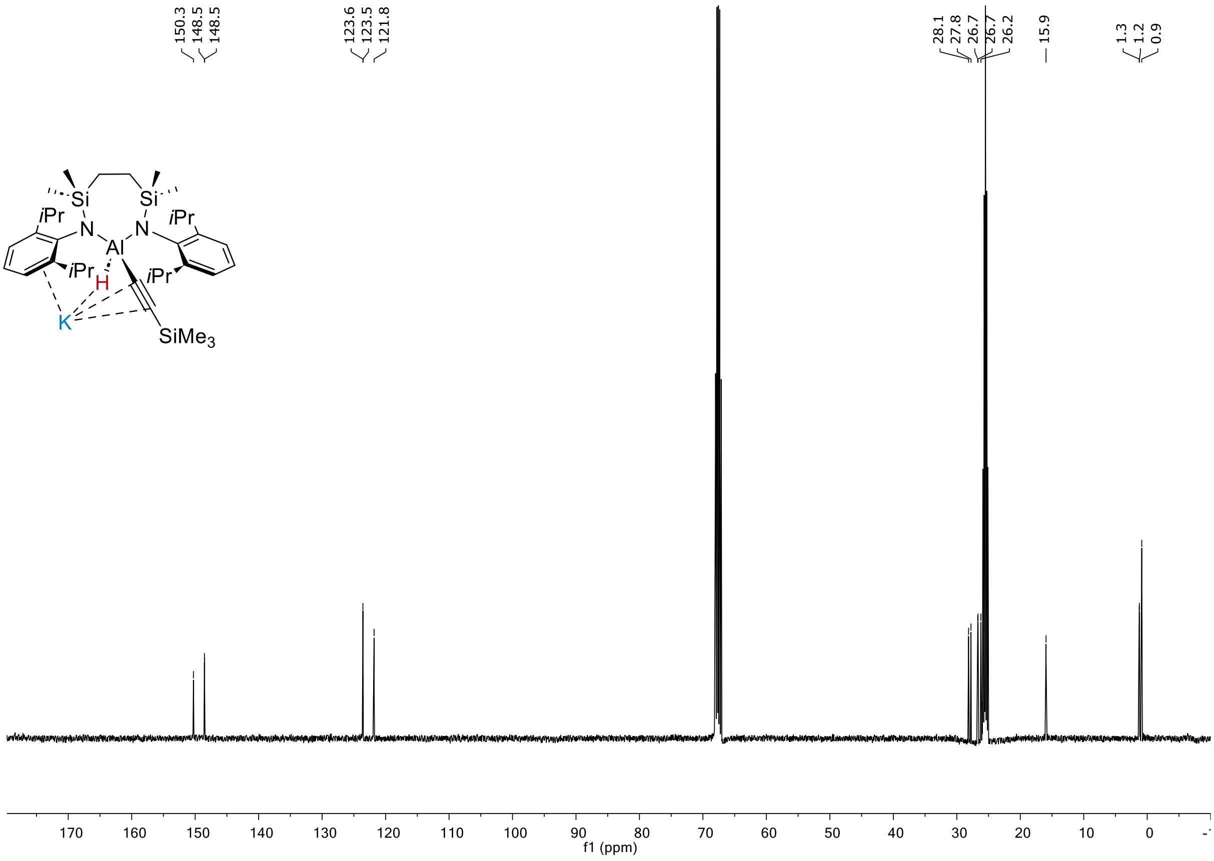

Figure S5: ^{1}H-^{13}C HSQC trace of **2**.

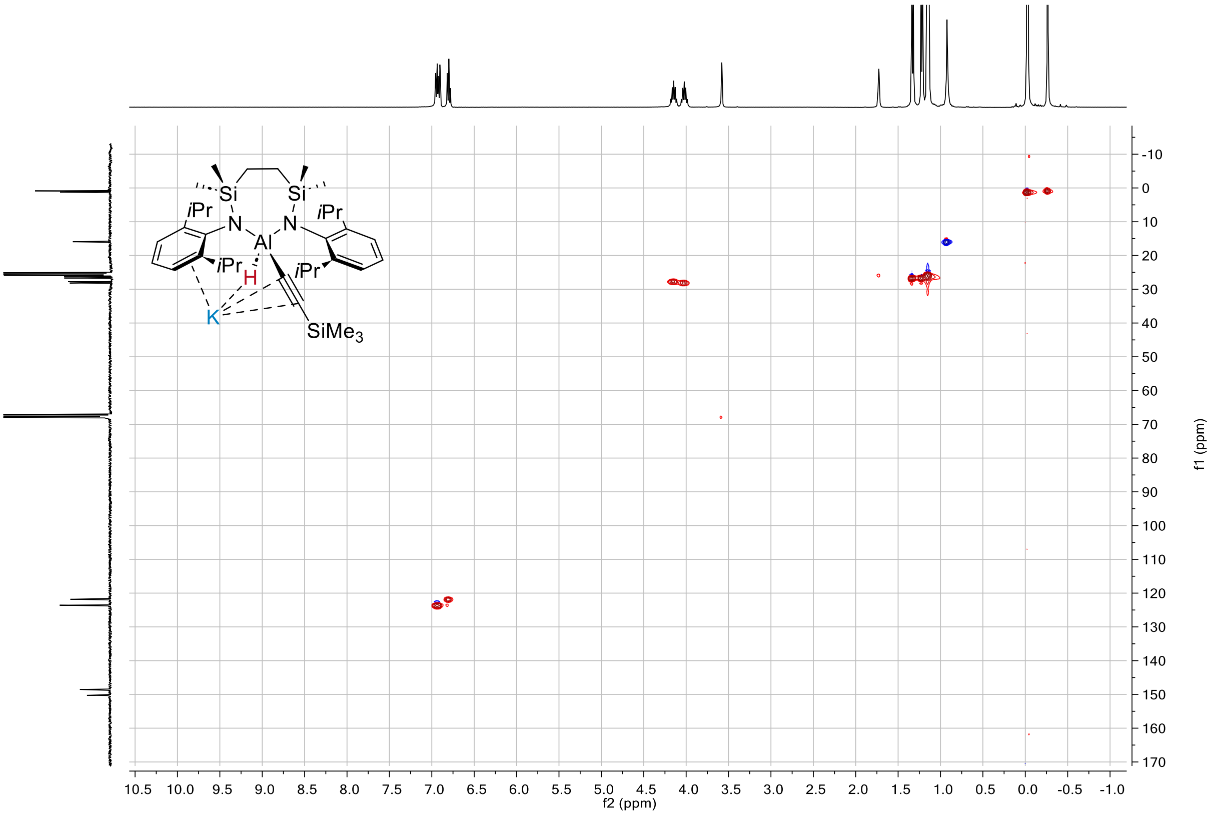

Figure S6: ^{1}H-^{13}C HMBC trace of **2**.

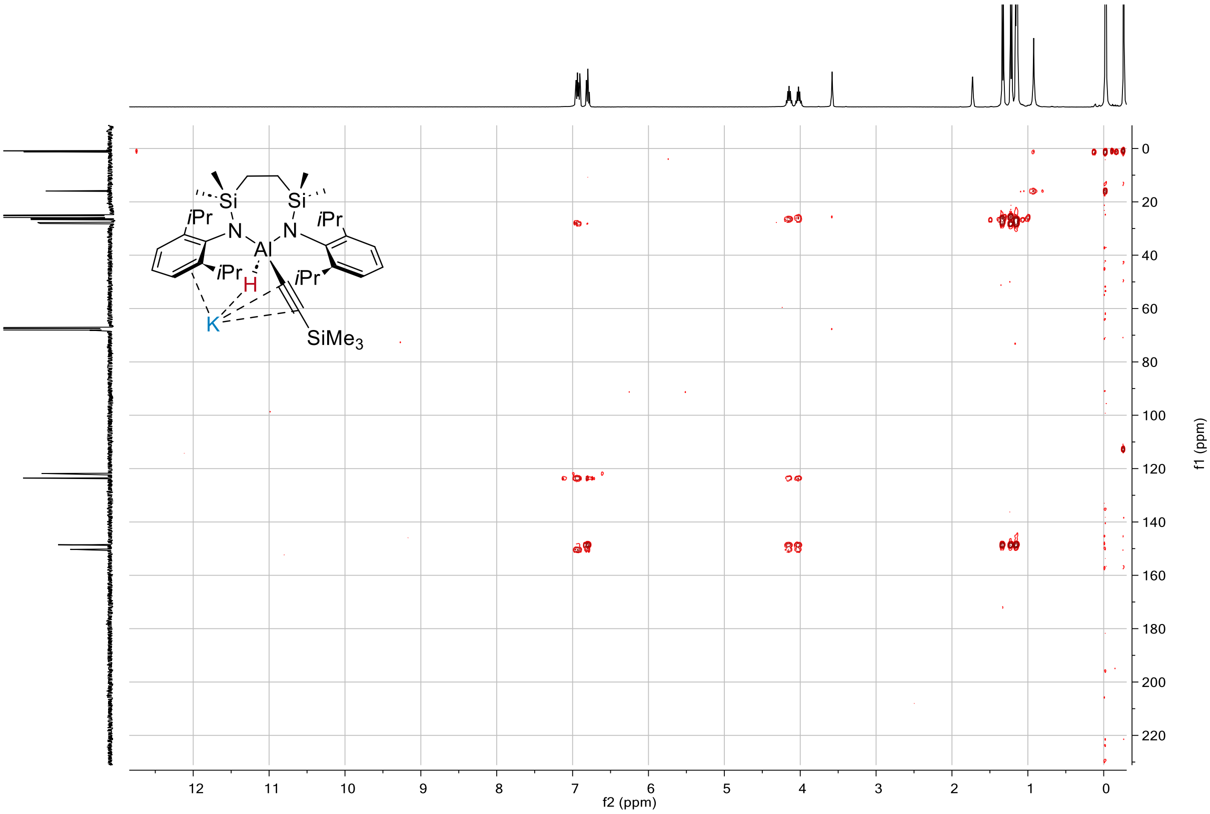

Figure S7: ^{1}H NMR (500 MHz, 298 K, d_8-THF) spectrum of **3**.

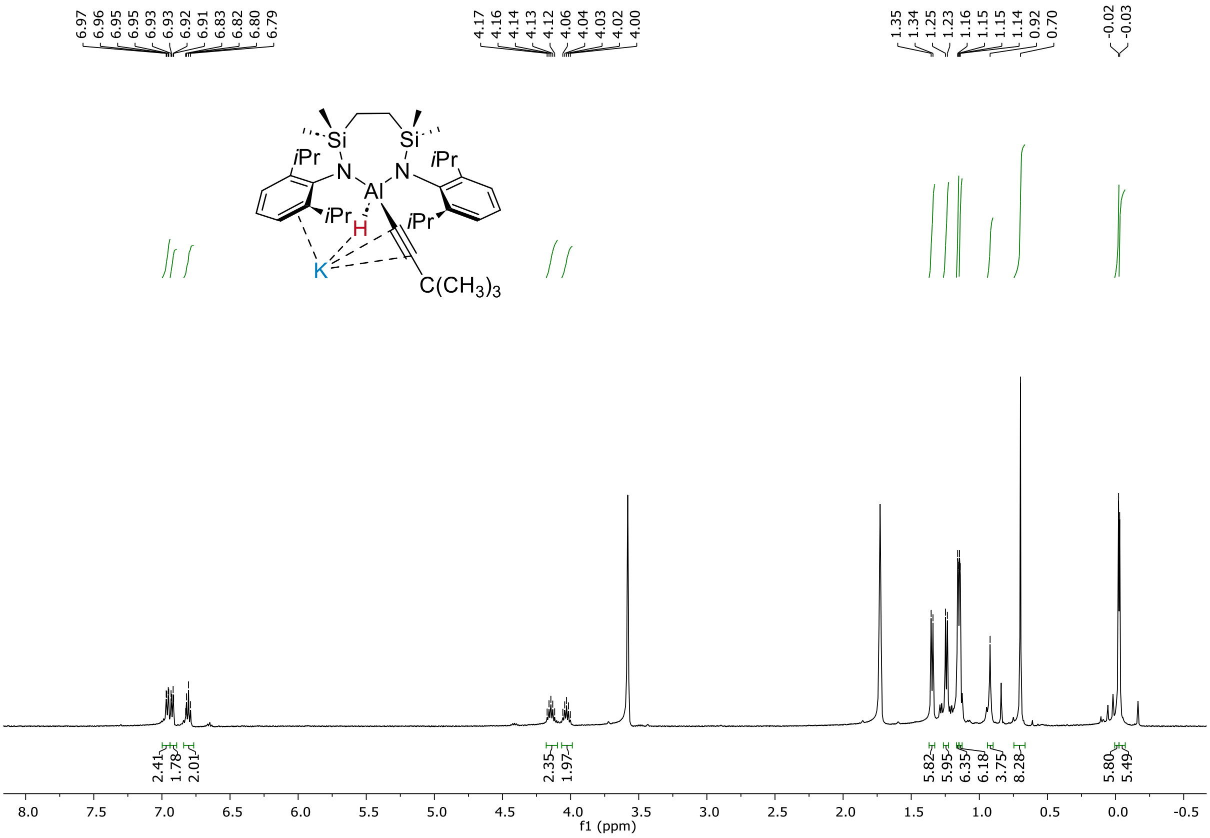

Figure S8: ^{13}C{^{1}H} NMR NMR (126 MHz, 298 K, d_8-THF) spectrum of **3**.

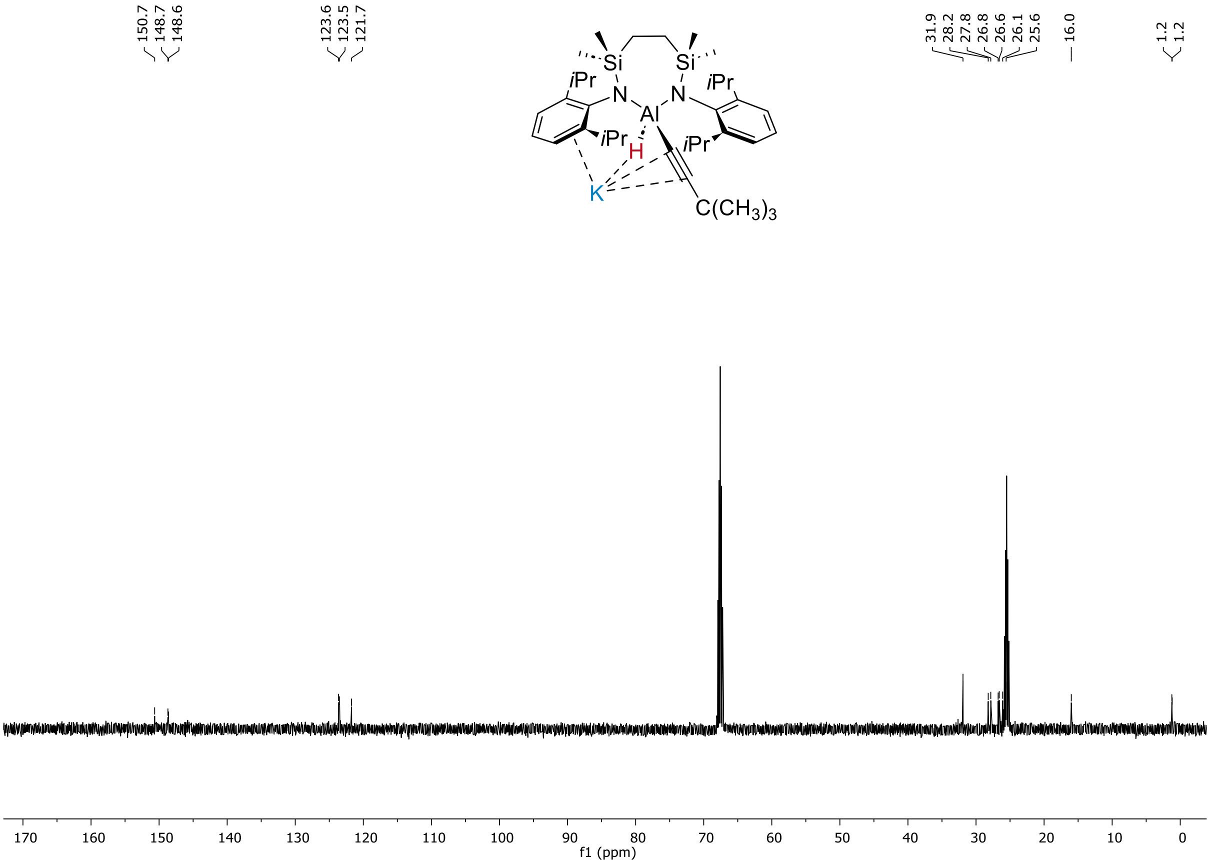

Figure S9: ^{1}H-^{13}C HSQC trace of **3**.

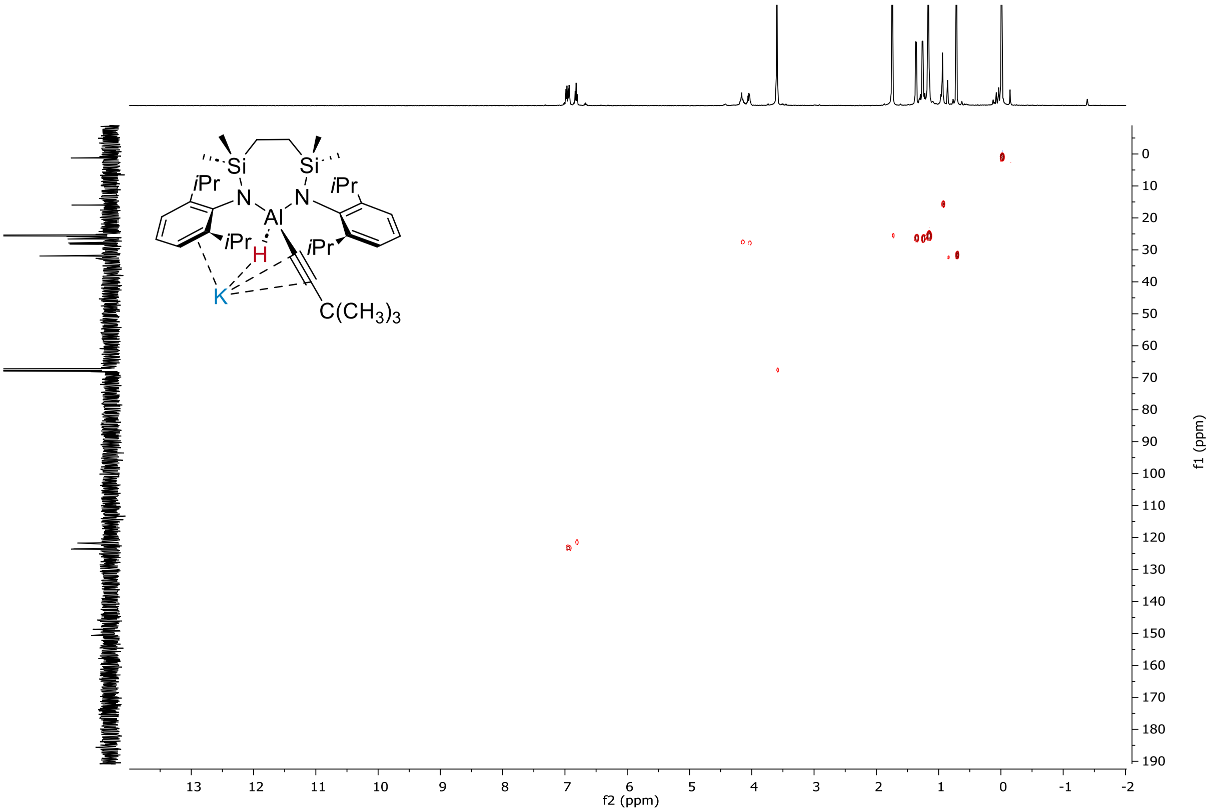

Figure S10: ^{1}H NMR (400 MHz, 298 K, d_8-THF) spectrum of **4**.

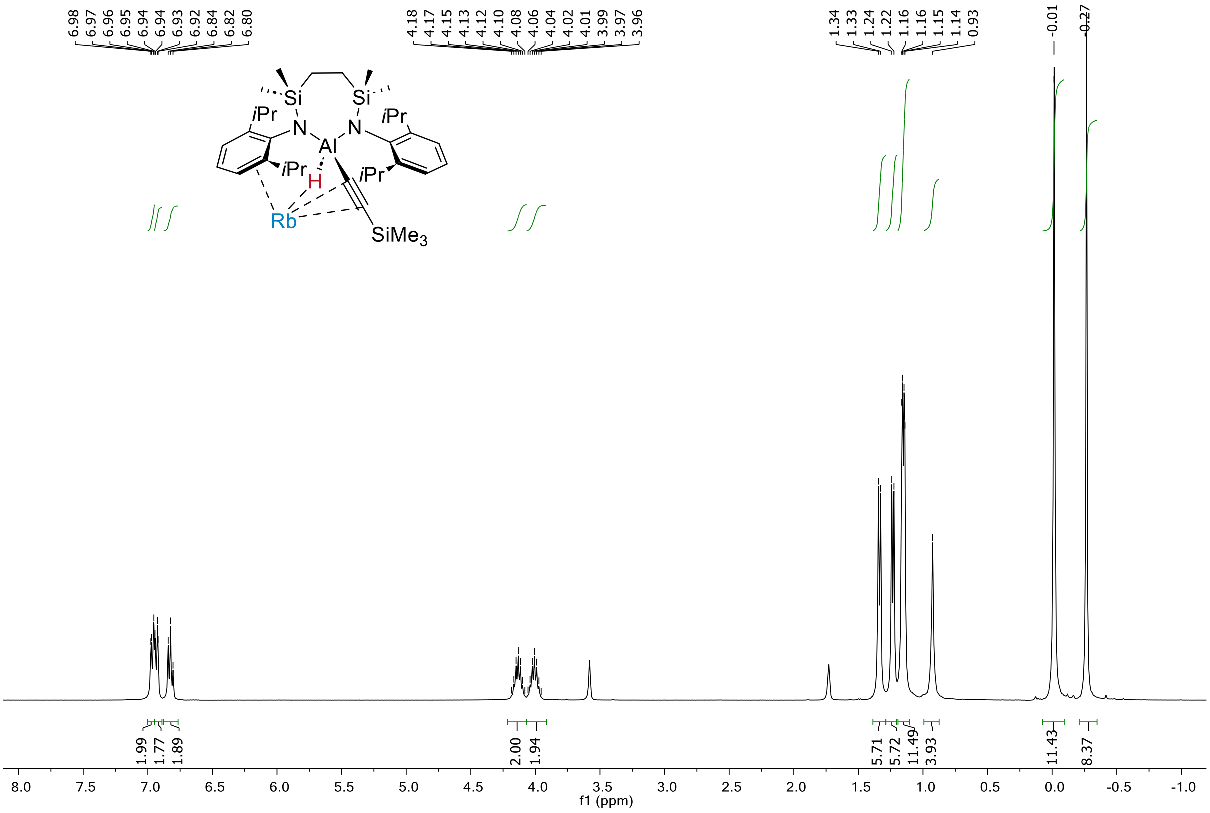

Figure S11: ^{13}C{^{1}H} NMR (101 MHz, 298 K, d_8-THF) spectrum of **4**.

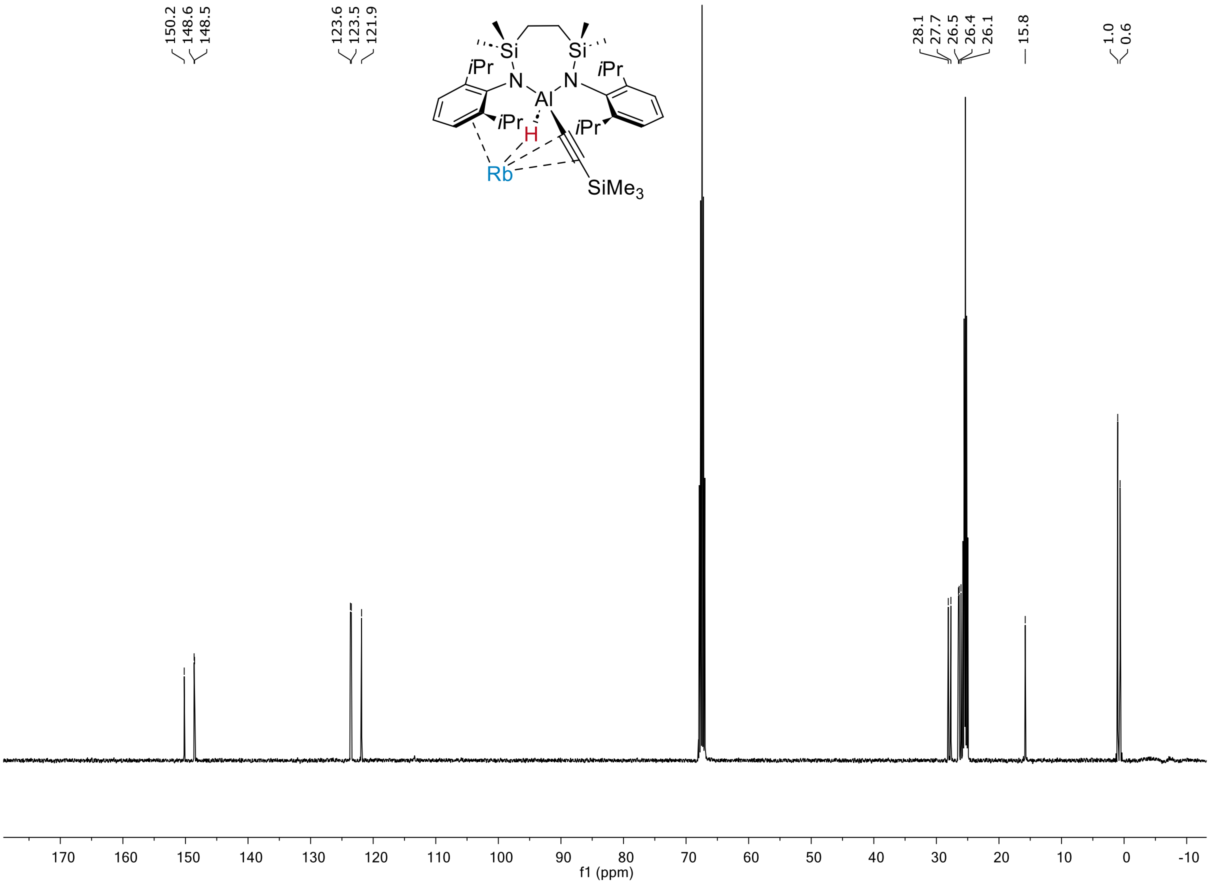

Figure S12: ^{1}H-^{13}C HSQC trace of **4.**

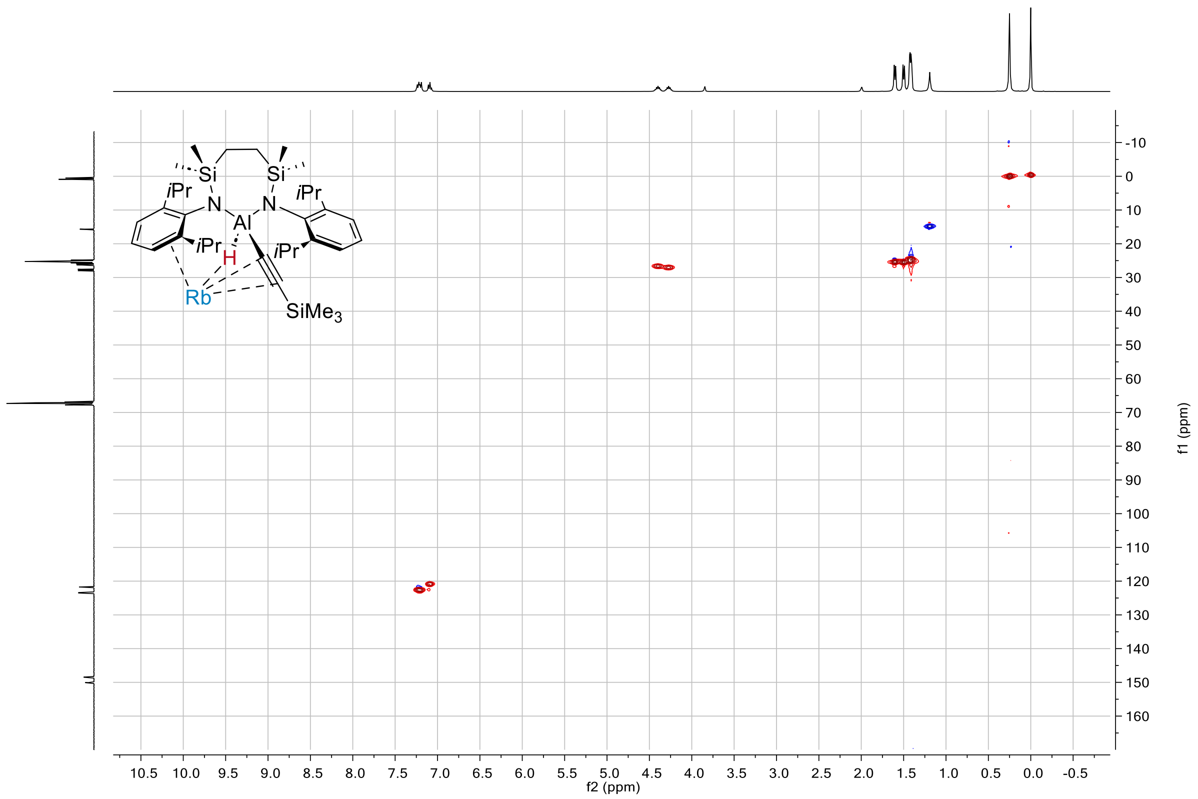

Figure S13: ^{1}H-^{13}C HMBC trace of **4**.

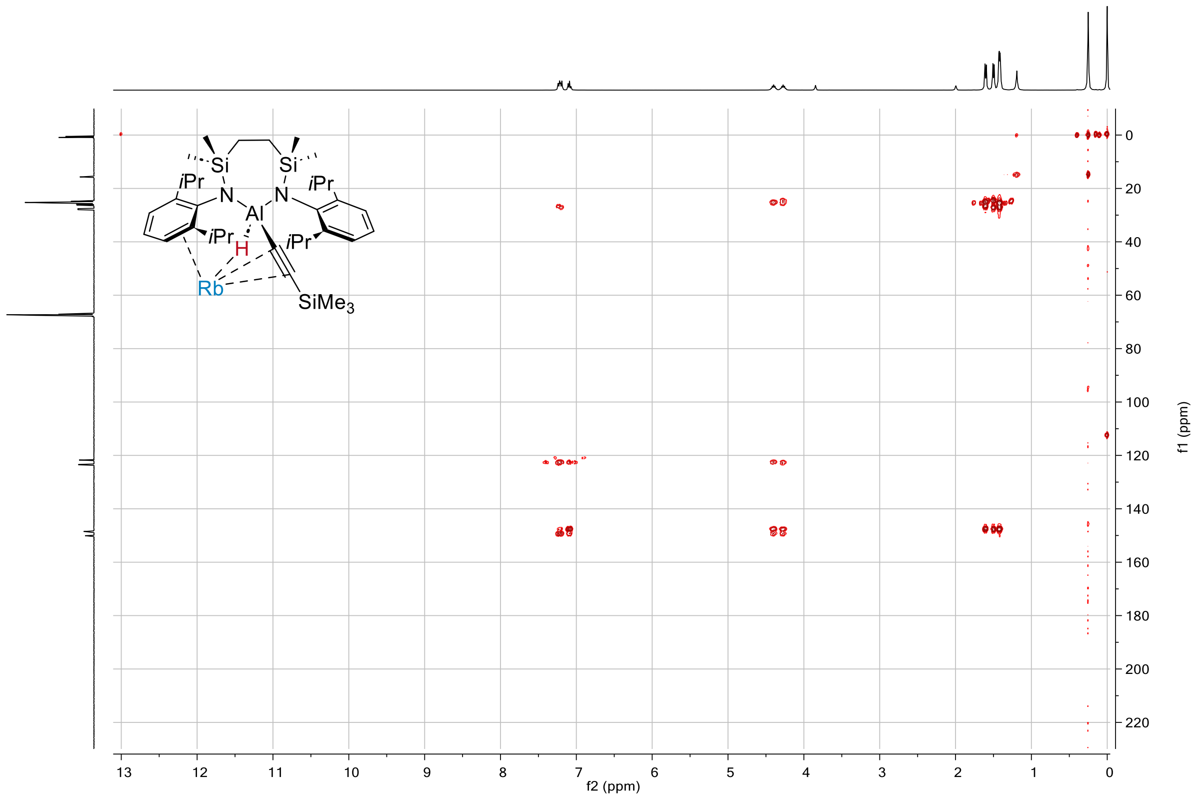

Figure S14: ^{1}H NMR (400 MHz, 298 K, d_8-THF) spectrum of **5**; *toluene.

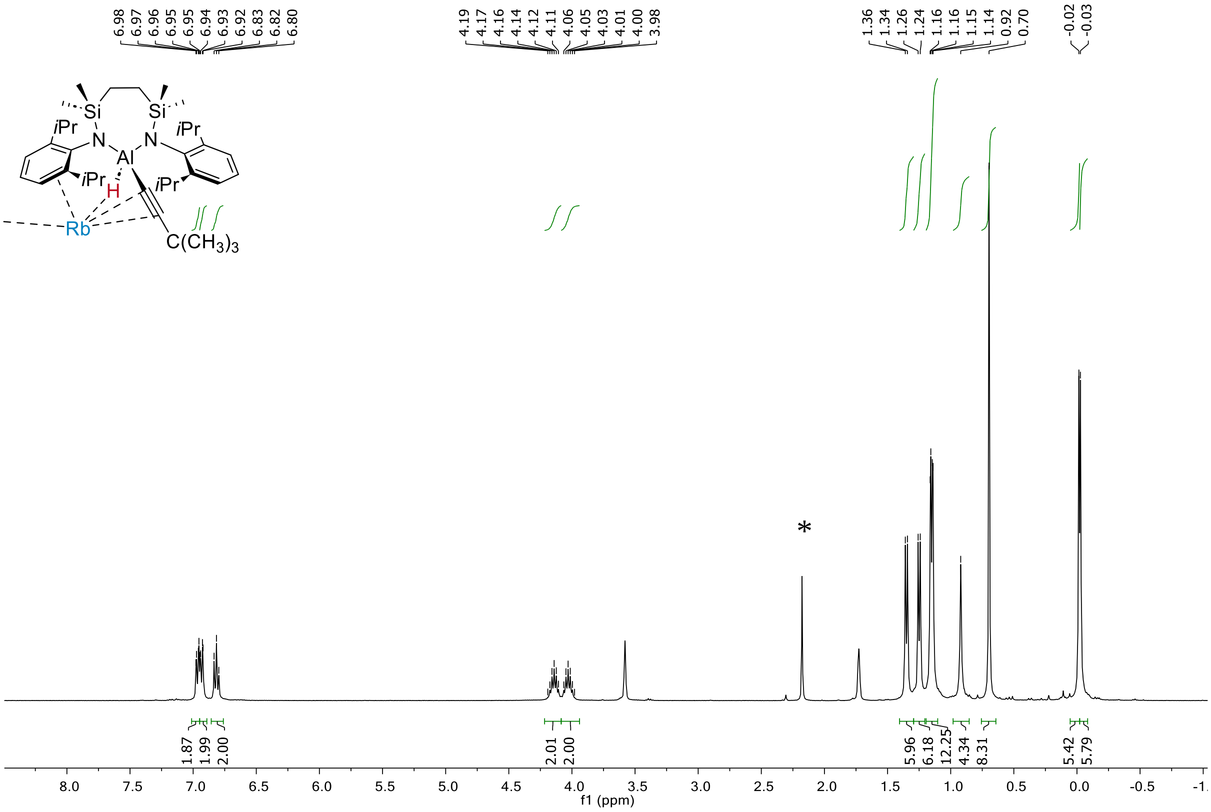

Figure S15: ^{13}C{^{1}H} NMR (101 MHz, 298 K, d_8-THF) spectrum of **5**.

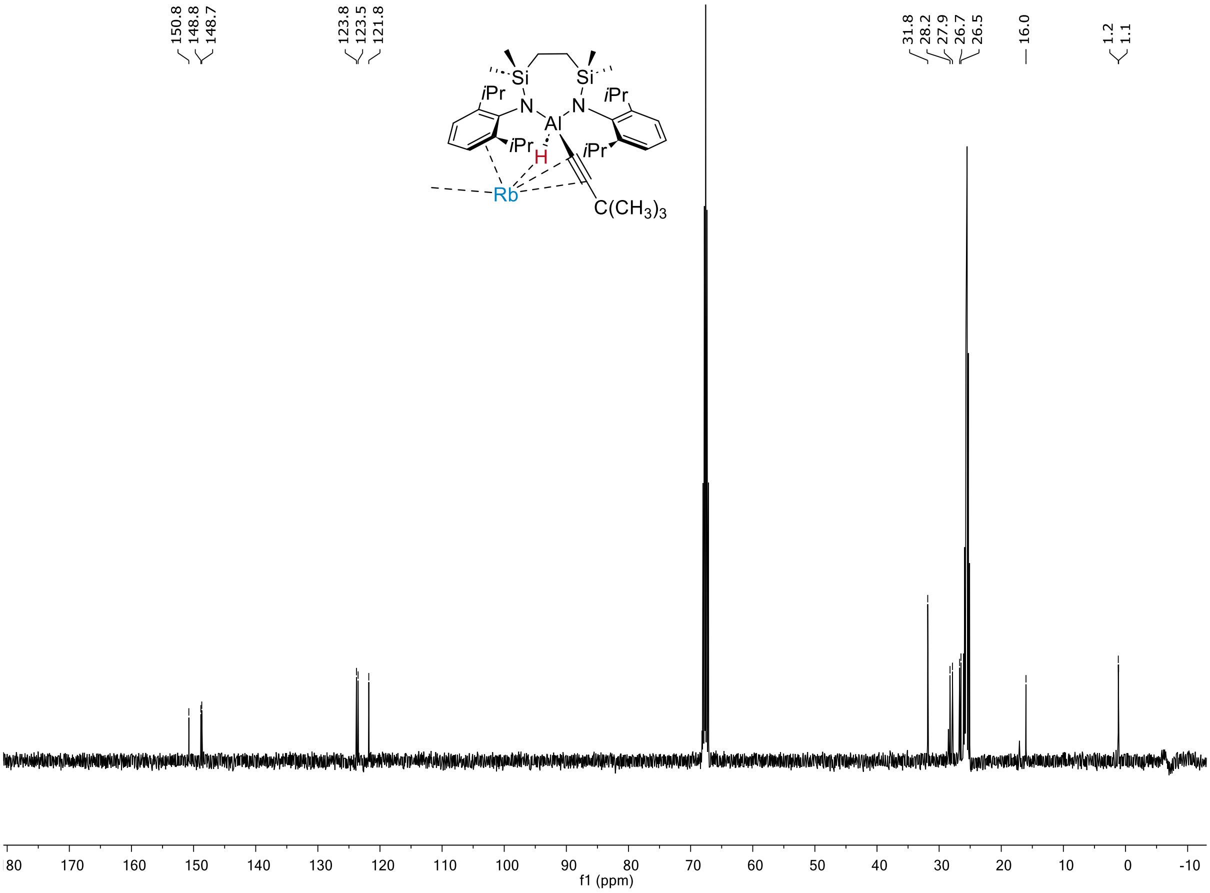

Figure S16: ^{1}H-^{13}C HSQC trace of **5**.

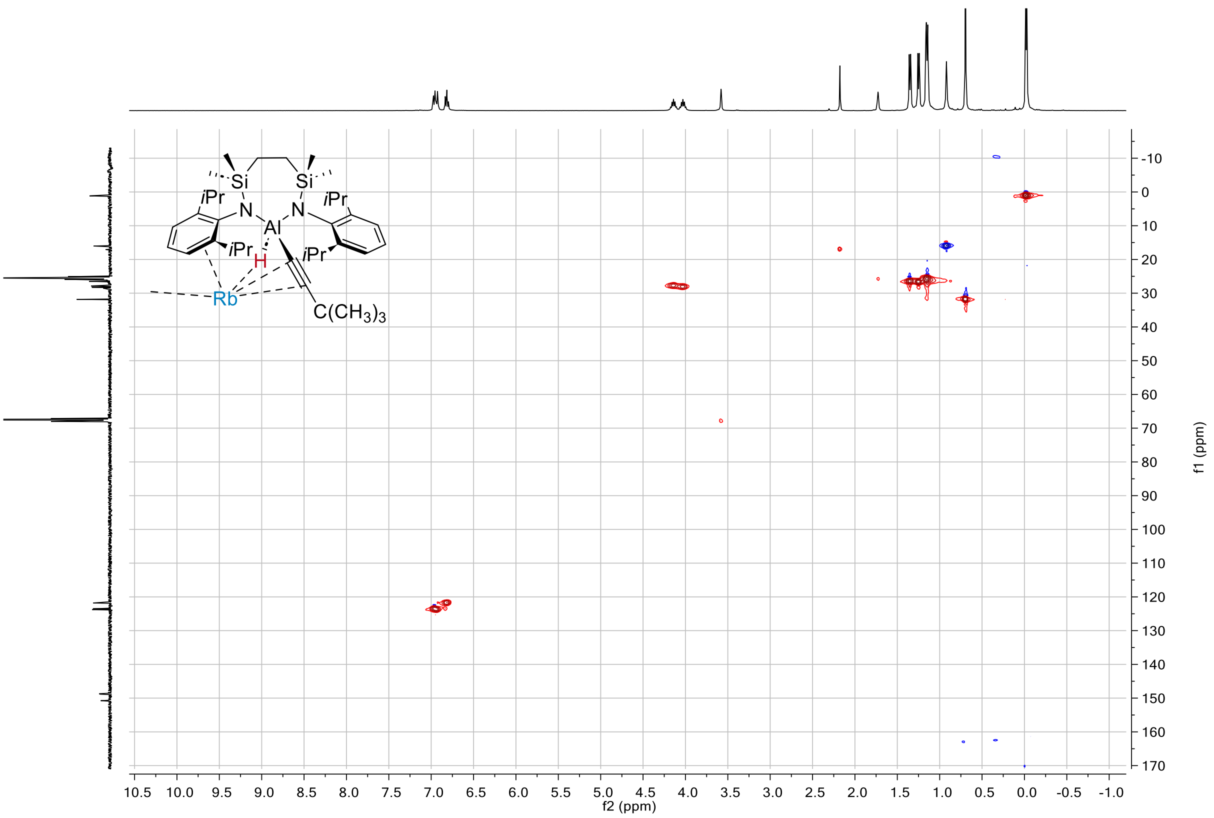

Figure S17: ^{1}H-^{13}C HMBC trace of **5**.

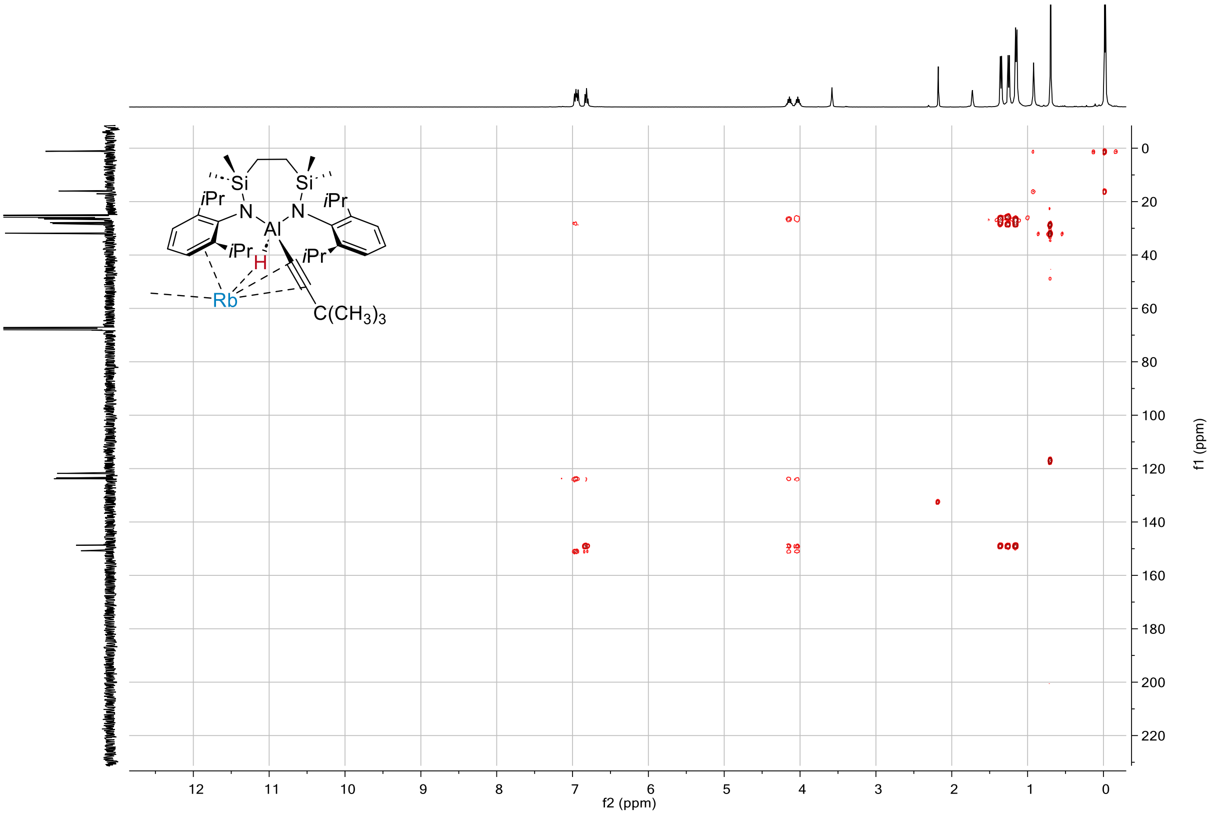

Figure S18: ^{1}H NMR (500 MHz, 298 K, Benzene-d_6) spectrum of **6**.

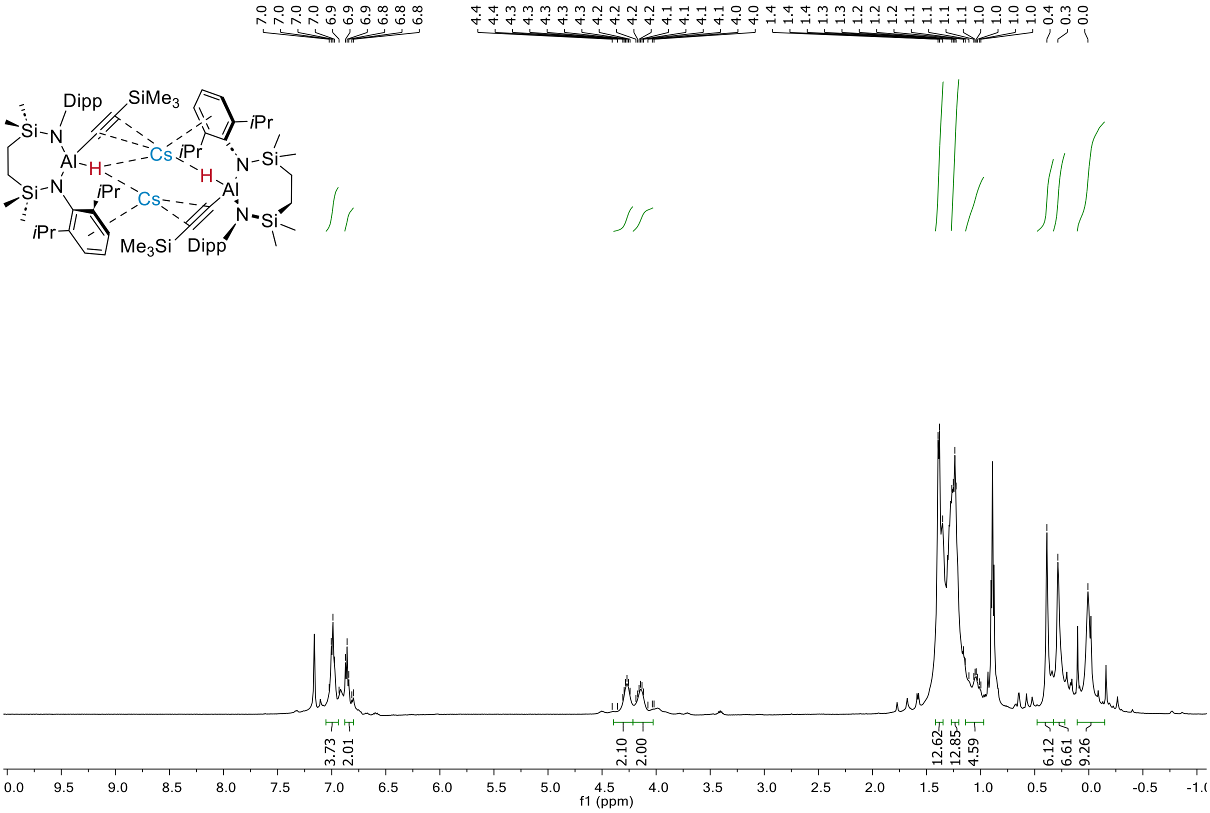

Figure S19: ^{13}C{^{1}H} NMR (126 MHz, 298 K, Benzene-d_6) spectrum of **6**.

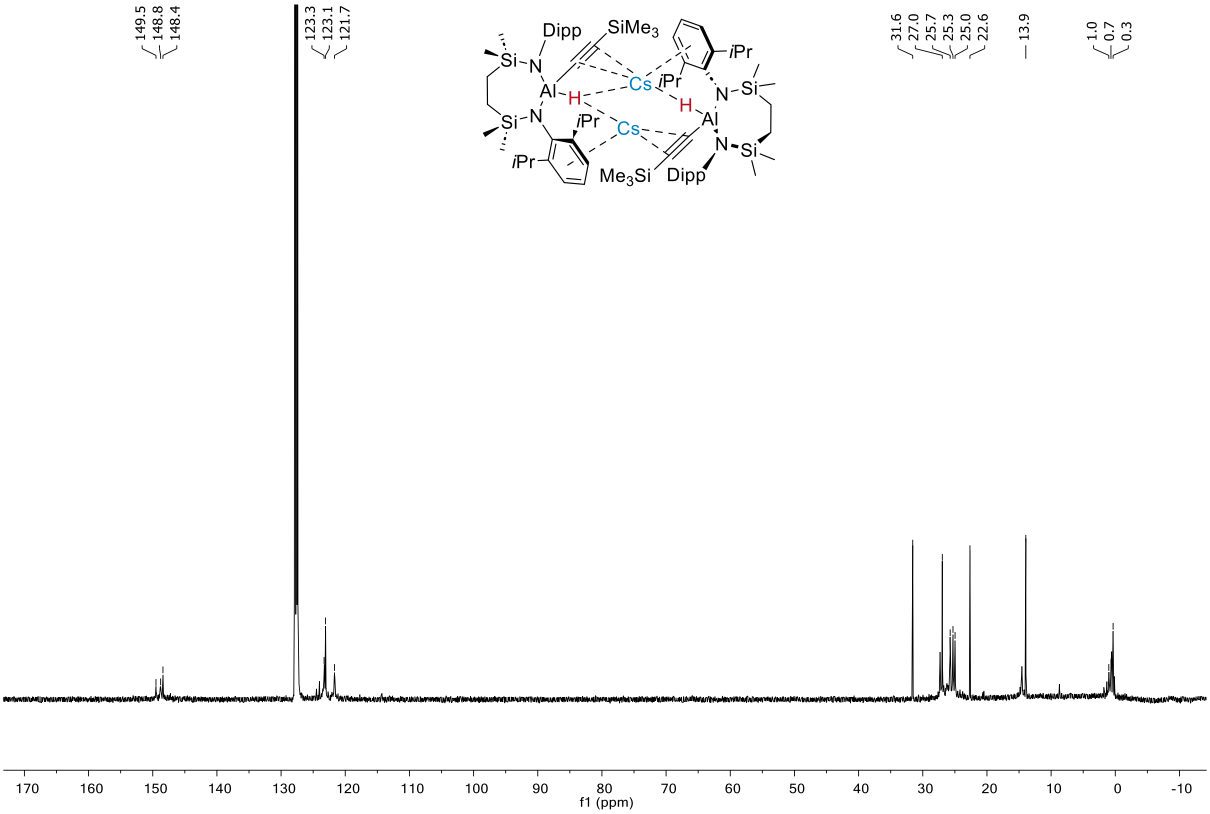

Figure S20: ^{1}H NMR (400 MHz, 298 K, d_8-THF) spectrum of **7**.

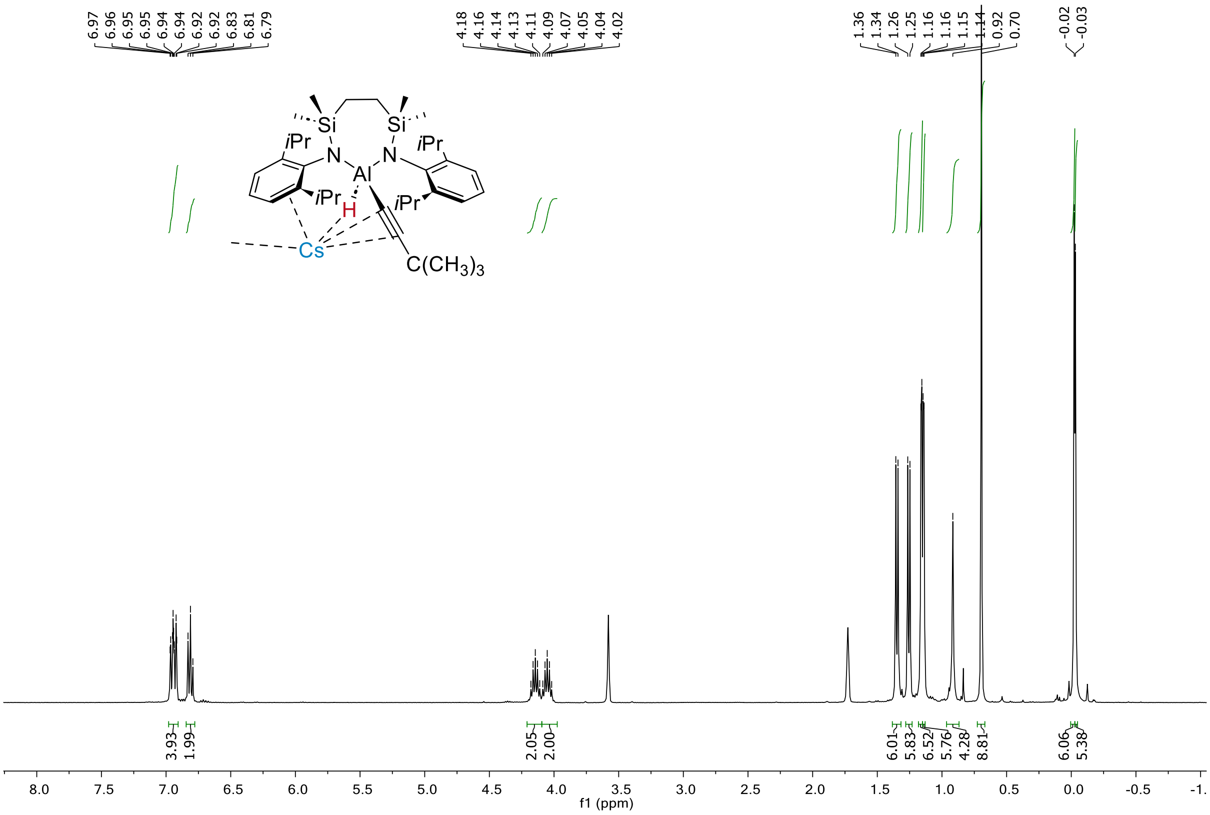

Figure S21: ^{13}C{^{1}H} NMR (101 MHz, 298 K, d_8-THF) spectrum of **7**.

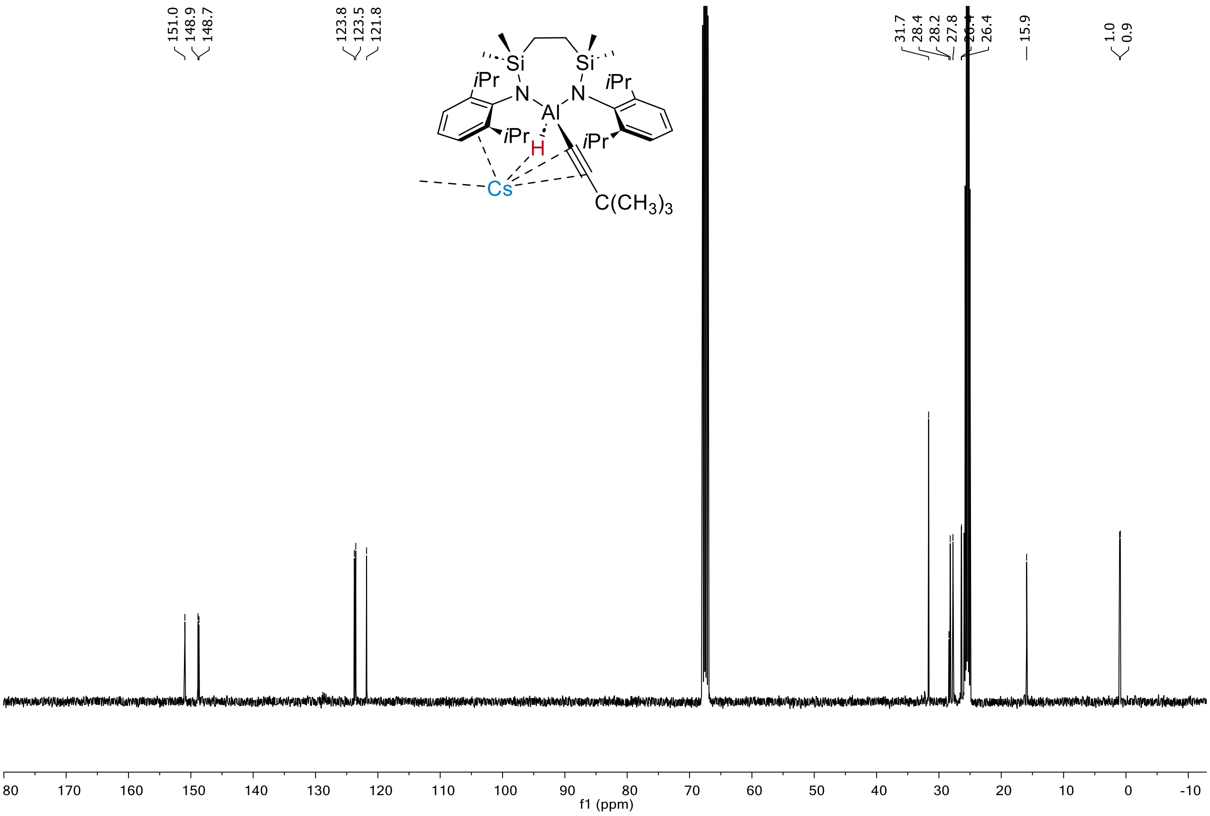

Figure S22: ^{1}H-^{13}C HSQC trace of **7**.

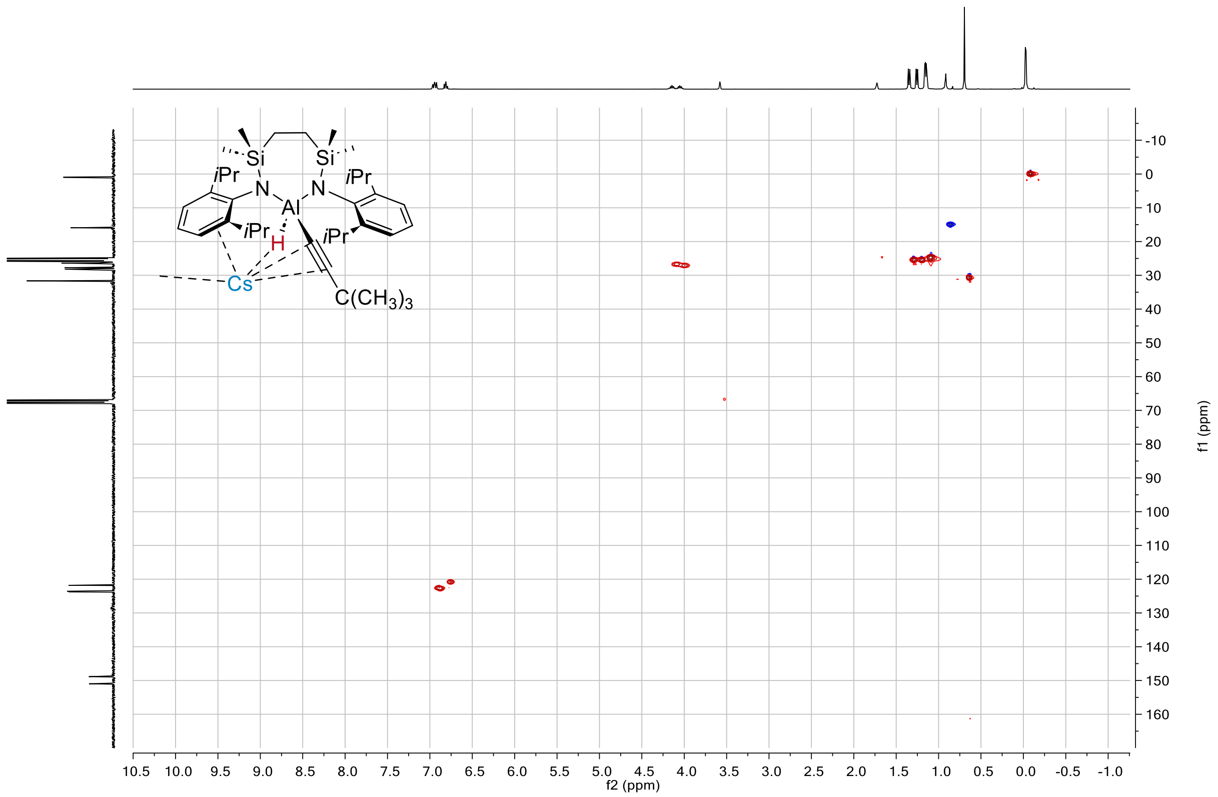

Figure S23: ^{1}H-^{13}C HMBC trace of **7**.

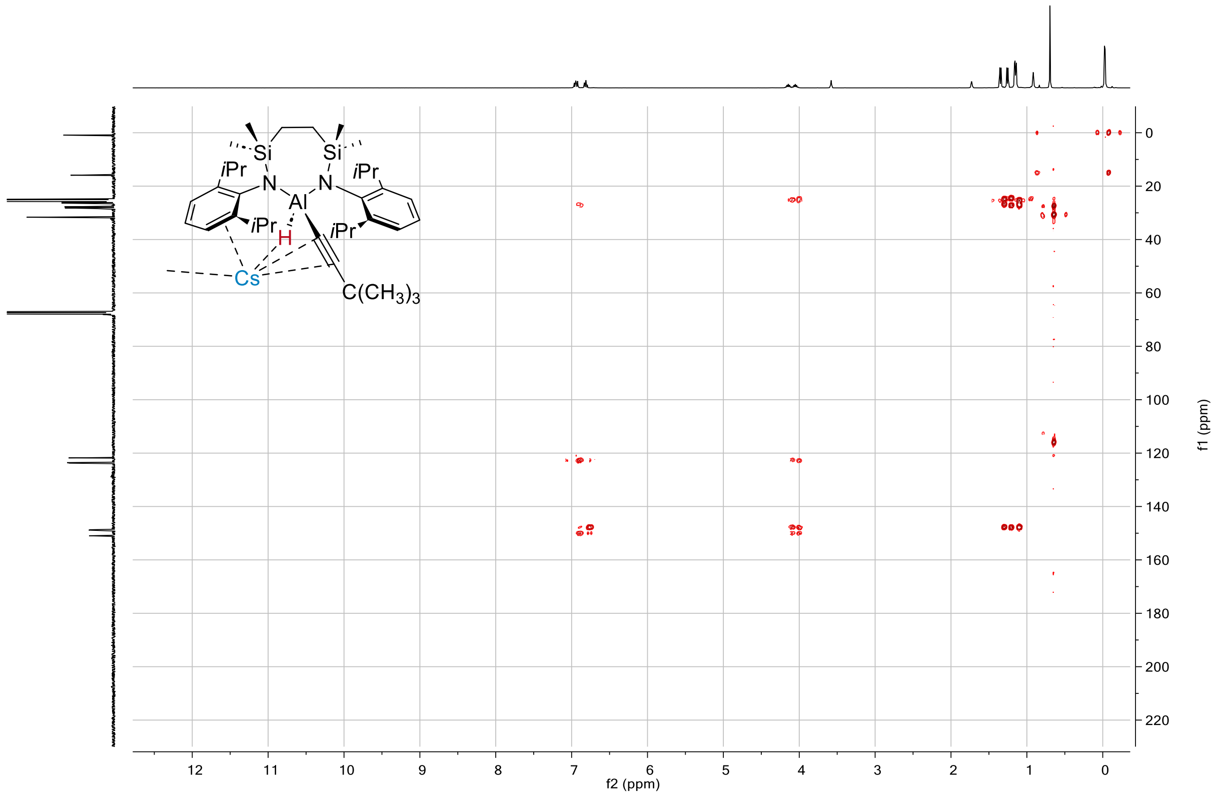

X-ray crystallography

Data for all structures barring **5** were obtained using an Agilent SuperNova instrument and a Cu-Kα source. Data for the outlier were obtained using an an Agilent Xcalibur instrument and Mo-Kα radiation. All experiments were conducted at 150 K and all structures were solved using SHELXT.[1] Refinements were effected using SHELXL[2] via the Olex2[3] interface. Convergences were generally straightforward. Where disorder prevailed, appropriate distance and ADP restraints were included, in these regions, to assist convergence. Only additional, noteworthy, points follow.

The asymmetric unit in the structure of **1** comprises one molecule of the aluminium/potassium complex, one full molecule of toluene and a toluene moiety with half site-occupancy. The latter (for which the phenyl ring was treated as a rigid hexagon in refinement) is proximate to a crystallographic inversion centre and is disordered with itself. The hydrides were located and refined freely, with fixed U_{iso} values.

In **2**, the asymmetric unit comprises a monomer which gives rise, the gross structure to 1-D polymers parallel to the *a*-axis. The hydrides were located and refined freely. Methyl hydrogens implicated in potassium-bound interactions were also located and, in these instances, each refined at a distance of 0.98 Å from the relevant parent atom.

The asymmetric unit in **3** also comprises a monomer which generates 1-D polymers in the gross structure. The aluminium bound hydrogen was located and refined without restraints, while those attached to C18 were located and refined at a distance of 0.96 Å from the parent atom.

Once again, a monomer containing two rubidium centres constitutes the asymmetric unit in **4**, and the gross structure is dominated by 1-D polymers. H1 and H2 were located and refined without restraints.

1-D polymers also arise from the asymmetric unit in **5.** The aluminium bound hydrogen was located and refined without restraints. While all remaining hydrogens were included at calculated positions, those with potential contact to the rubidium centres (H14A, H14B, H14C) were refined with free U_{iso} values in order to afford additional credibility regarding their locations.

Half of a dimer constitutes the asymmetric unit in **6**. The remainder of the molecule arises *via* crystallographic inversion symmetry. The caesium centre was treated for 89:11 disorder. H1

was located and refined with a riding U_{iso} value but without positional restraints. The reason for using a riding U_{iso} for H1 was that the model had difficulty in converging without same.T here is potential for an intermolecular interaction between the minor component of the caesium centre, and a hydrogen atom attached to C17.

Finally, in **7** the asymmetric unit comprises a monomer which generates 1-D polymers in the gross structure, which propagate along the *b*-axis. While all carbon-bound hydrogens were included at calculated positions, those with potential contact to the caesium centre (C14, C26) were refined with free U_{iso} values. H1 was located and refined without restraints. The highest residual electron density is at non-chemically significant distances from the caesium centre. Efforts to model disorder in this region were abandoned as the arising models afforded little evidence for the veracity of same.

Crystallographic data for all compounds have been deposited with the Cambridge Crystallographic Data Centre as supplementary publications CCDC 2365673-2365679 for **1**, **2**, **3**, **4**, **5**, **6** and **7**, respectively. Copies of these data can be obtained free of charge on application to CCDC, 12 Union Road, Cambridge CB2 1EZ, UK [fax(+44) 1223 336033, e-mail: deposit@ccdc.cam.ac.uk.

Table S1: Data collection and refinement parameters for **1** – **4**.

Manuscript Identifier	**1**	**2**	**3**	**4**
Empirical formula	$C_{92.5}H_{136}Al_2K_2N_4Si_4$	$C_{35}H_{60}AlKN_2Si_3$	$C_{36}H_{60}AlKN_2Si_2$	$C_{35}H_{60}AlN_2RbSi_3$
Formula weight	1548.56	659.20	643.12	705.57
Crystal system	triclinic	monoclinic	orthorhombic	monoclinic
Space group	*P*-1	$P2_1/c$	*Pbca*	$P2_1/c$
a/ Å	14.1666(2)	17.6602(2)	12.4668(1)	17.8892(2)
b/ Å	15.0600(2)	36.0245(5)	17.8660(2)	35.9477(4)
c/ Å	24.0306(3)	12.65629(13)	34.9717(6)	12.6575(1)
α/ °	100.769(1)	90	90	90
β/ °	101.146(1)	90.0844(9)	90	90.473(1)
γ/ °	108.542(1)	90	90	90
U/ $Å^3$	4596.01(11)	8051.94(17)	7789.31(17)	8139.45(14)
Z	2	8	8	8
ρ_{calc}/ g cm^{-3}	1.119	1.088	1.097	1.152
μ/ mm^{-1}	1.924	2.391	2.175	2.896
F(000)	1678.0	2864.0	2800.0	3008.0
Crystal size/ mm^3	0.214 × 0.128 × 0.113	0.223 × 0.143 × 0.095	0.233 × 0.189 × 0.15	0.187 × 0.125 × 0.079
2θ range for data collection/°	7.468 to 145.866	7.01 to 146.662	8.712 to 146.358	6.972 to 145.82
Index ranges	$-16 \le h \le 17$, $-18 \le k \le 18$, $-29 \le l \le 23$	$-21 \le h \le 21$, $-36 \le k \le 43$, $-15 \le l \le 15$	$-15 \le h \le 9$, $-21 \le k \le 21$, $-42 \le l \le 43$	$-19 \le h \le 22$, $-43 \le k \le 44$, $-15 \le l \le 15$
Reflections collected	65215	94632	103742	104501
Independent reflections, R_{int}	18224, 0.0242	15942, 0.0782	7762, 0.0791	16163, 0.0490
Data/restraints/parameters	18224/42/1022	15942/6/817	7762/0/409	16163/0/831
Goodness–of–fit on F^2	1.025	1.065	1.048	1.152
Final *R*1, *wR*2 [I>=2σ(I)]	0.0391, 0.1047	0.0874, 0.2186	0.0522, 0.1368	0.0826, 0.2121
Final *R*1, *wR*2 [all data]	0.0423, 0.1074	0.0975, 0.2302	0.0631, 0.1440	0.0847, 0.2130
Largest diff. peak/hole/ e $Å^{-3}$	1.51/–0.37	1.08/–0.63	0.55/–0.31	1.03/–0.59

Table S2: Data collection and refinement parameters for **5** – **7**.

Manuscript Identifier	**5**	**6**	**7**
Empirical formula	$C_{36}H_{60}AlN_2RbSi_2$	$C_{35}H_{60}AlCsN_2Si_3$	$C_{36}H_{60}AlCsN_2Si_2$
Formula weight	689.49	753.01	736.93
Crystal system	orthorhombic	monoclinic	orthorhombic
Space group	*Pbca*	$P2_1/n$	*Pbca*
a/ Å	12.4855(3)	13.4979(1)	12.5525(2)
b/ Å	18.0186(4)	17.8552(2)	18.1451(3)
c/ Å	35.1235(12)	16.6691(1)	35.1493(7)
α/ °	90	90	90
β/ °	90	98.946(1)	90
γ/ °	90	90	90
U/ Å^3	7901.8(4)	3968.51(6)	8005.8(2)
Z	8	4	8
ρ_{calc}/ g cm^{-3}	1.159	1.260	1.223
μ/ mm^{-1}	1.361	8.533	8.172
F(000)	2944.0	1576.0	3088.0
Crystal size/ mm^3	0.356 × 0.258 × 0.232	0.109 × 0.089 × 0.057	0.361 × 0.156 × 0.107
2θ range for data collection/°	6.04 to 59.15	7.304 to 145.95	8.656 to 146.96
Index ranges	$-16 \le h \le 16$, $-23 \le k \le 24$, $-48 \le l \le 48$	$-13 \le h \le 16$, $-22 \le k \le 22$, $-20 \le l \le 20$	$-15 \le h \le 9$, $-22 \le k \le 22$, $-43 \le l \le 42$
Reflections collected	71953	52837	32583
Independent reflections, R_{int}	10659, 0.0540	7909, 0.0561	7839, 0.0542
Data/restraints/parameters	10659/0/401	7909/6/406	7839/0/412
Goodness–of–fit on F^2	1.060	1.047	1.027
Final R1, wR2 [I>=2σ(I)]	0.0455, 0.0846	0.0404, 0.1029	0.0617, 0.1617
Final R1, wR2 [all data]	0.0737, 0.0926	0.0428, 0.1051	0.0708, 0.1744
Largest diff. peak/hole/ e Å^{-3}	0.33/–0.32	1.53/–0.70	1.47/–0.64

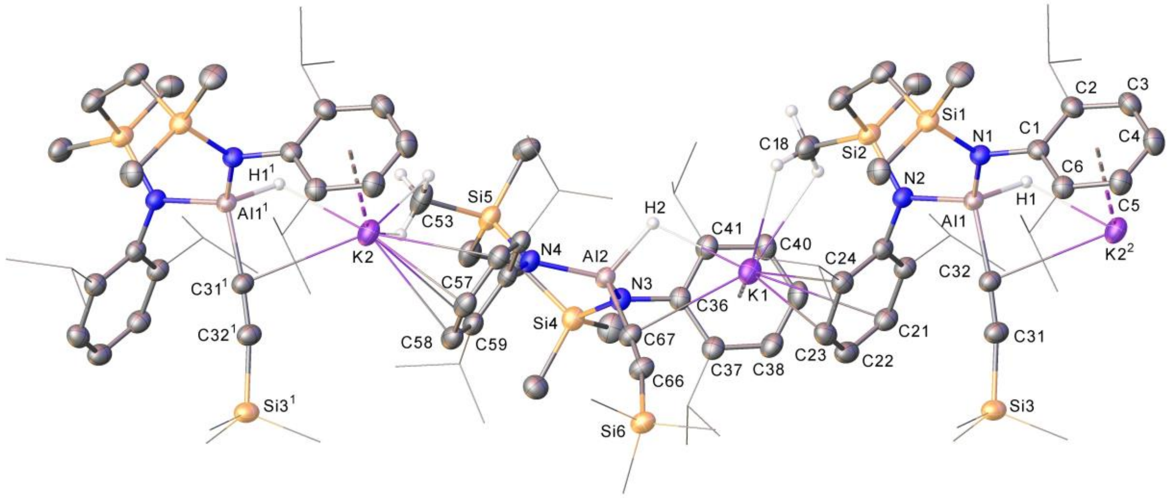

Figure S24: Plot depicting the structure of **2**. Ellipsoids are depicted at 30% probability. Hydrogen atoms (H1, H2 and those attached to C18 and C53 excepted) have been omitted and peripheral substituents are depicted as wireframes, for clarity. Symmetry operations: ^{1} 1 + *x*, *y*, *z*; ^{2} –1 + *x*, *y*, *z*.

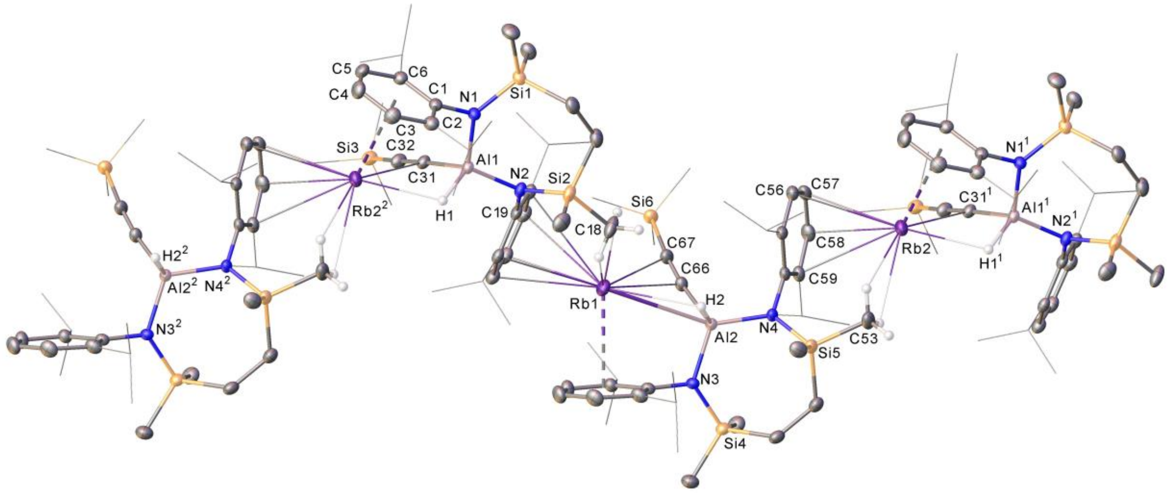

Figure S25: Plot depicting the structure of **4**. Ellipsoids are depicted at 30% probability. Hydrogen atoms (H1, H2 and those attached to C18 and C53 excepted) have been omitted and peripheral substituents are depicted as wireframes, for clarity. Symmetry operations: ^{1} 1 + *x*, *y*, *z*; ^{2} –1 + *x*, *y*, *z*.

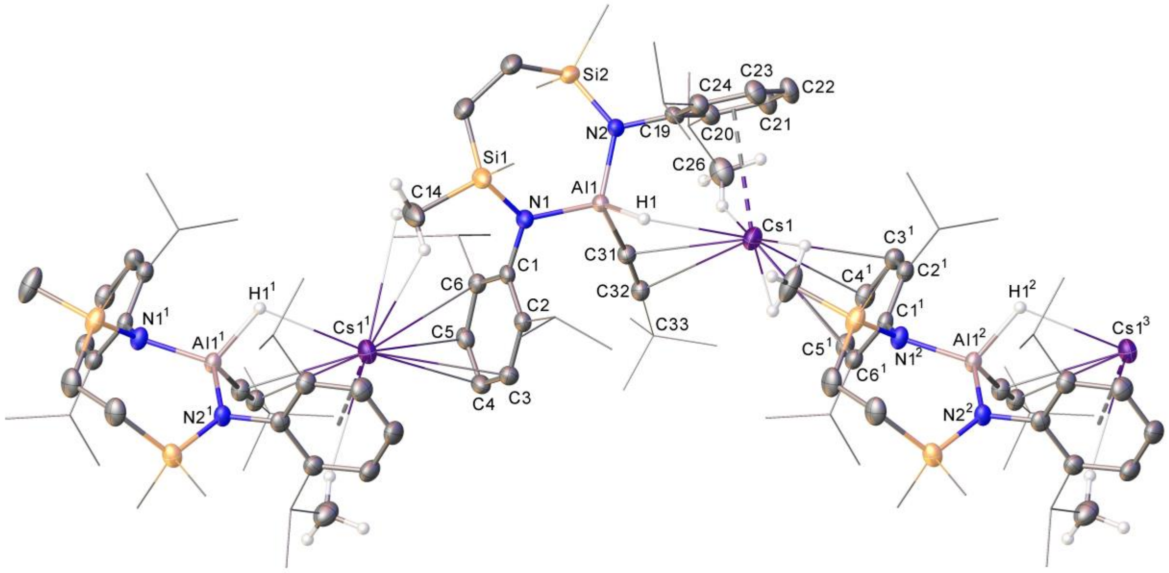

Figure S26: Plot depicting the structure of **7**. Ellipsoids are depicted at 30% probability. Hydrogen atoms (H1 and those attached to C14 and C26 excepted) have been omitted and peripheral substituents are depicted as wireframes, for visual ease. Symmetry operations: [1] 3/2 – *x*, –½ + *y*, *z*; [2] *x*, –1 + *y*, *z*; [3] 3/2 – *x*, ½ + *y*, *z*.

2. Computational Supporting Information

2.1. Computational Details

DFT calculations were run with Gaussian 16 (C.01).[4] The Na, Al, Si, K, Cs and Rb centres were described with the Stuttgart RECPs and associated basis sets,[5] and 6-31G** basis sets were used for all other atoms (BS1).[6] A polarization function was also added to Al (ζd = 0.190), Si (ζd = 0.284), K (ζd = 1.000), Rb (ζd = 0.491) and Cs (ζd = 0.306). Initial BP86[7] optimizations were performed using the 'grid = ultrafine' option, with all stationary points being fully characterized via analytical frequency calculations as minima (all positive eigenvalues) or transition states (one negative eigenvalue). All energies were recomputed with a larger basis set featuring 6-311++G** basis sets on all atoms, with the exception of Rb and Cs, where def2-TVZP was used (BS2). Corrections for the effect of benzene (ε = 2.2706) solvent were run using the polarizable continuum model and BS1, using the keyword "scrf=benzene" within Gaussian.[8] Single-point dispersion corrections to the BP86/BS1 results employed Grimme's D3 parameter set with Becke-Johnson damping as implemented in Gaussian.[9] Note, the structures and energies of $\mathbf{A}^{\mathbf{K}}$, $\mathbf{A}^{\mathbf{Rb}}$ and $\mathbf{A}^{\mathbf{Cs}}$ are already reported in a previous study.[10]

2.2. Breakdown of energy contributions

The following table details the evolution of the relative energies as the successive corrections to the initial SCF energy are included. Terms used are:

$\mathbf{\Delta E_{BSI}}$	SCF energy computed with the BP86 functional with BS1
$\mathbf{\Delta H_{BSI}}$	Enthalpy at 0 K with BS1
$\mathbf{\Delta G_{BSI}}$	Free energy at 298.15 K and 1 atm with BS1
$\mathbf{\Delta G_{BSI/C_6H_6}}$	Free energy corrected for C_6H_6 solvent with BS1
$\mathbf{\Delta G_{BSI/C_6H_6+D3BJ}}$	Free energy corrected for C_6H_6 and dispersion effects (D3BJ) with BS1
$\mathbf{\Delta E_{BS2}}$	SCF energy computed with the BP86 functional with BS2
$\mathbf{\Delta G_{C_6H_6}}$	Free energy corrected for BS2, D3BJ and C_6H_6 solvent

In each case the final data used in the main article are highlighted in bold.

Table S3: Energies breakdown in kcal mol^{-1} for monomeric mechanisms BP86-D3BJ(benzene)/BS2//BP86/BS1.

**Species**	ΔE_{BSI}	ΔH_{BSI}	ΔG_{BSI}	$\Delta G_{BSI/C_6H_6}$	$\Delta G_{BSI/C_6H_6+D3BJ}$	ΔE_{BS2}	ΔG_{bnz}
**A^K**	0.0	0.0	0.0	0.0	0.0	0.0	**0.0**
**B^K**	-0.6	0.5	6.2	6.5	2.4	0.6	**3.6**
**$TS(B\text{-}C)^K$**	17.1	14.3	25.9	26.1	9.3	20.2	**12.5**
**C^K**	-0.7	-1.6	4.3	2.3	1.9	-2.0	**0.6**
**$TS(C\text{-}D)^K$**	2.0	0.7	11.0	10.6	-2.4	2.6	**-1.7**
**D^K**	-34.1	-34.2	-19.0	-18.4	-42.1	-30.6	**-38.6**
**E^K**	-34.9	-33.8	-12.6	-12.2	-37.9	-30.2	**-33.2**
**$TS(E\text{-}F)^K$**	-16.5	-19.1	7.8	7.4	-23.5	-10.9	**-17.9**
**F^K**	-33.7	-34.5	-14.0	-16.0	-37.3	-31.1	**-34.7**
**$TS(F\text{-}G)^K$**	-33.8	-34.8	-7.4	-7.2	-40.5	-29.4	**-36.1**
**G^K**	-68.4	-68.6	-38.0	-37.0	-81.1	-61.6	**-74.3**
**A^{Rb}**	0.0	0.0	0.0	0.0	0.0	0.0	**0.0**
**B^{Rb}**	-0.9	0.0	5.0	5.3	0.4	0.5	**1.9**
**$TS(B\text{-}C)^{Rb}$**	17.0	14.2	25.3	25.6	6.6	20.3	**9.9**
**C^{Rb}**	0.0	-0.8	4.5	2.7	2.4	-0.7	**1.7**
**$TS(C\text{-}D)^{Rb}$**	3.1	1.9	12.8	12.3	-3.1	4.3	**-1.9**
**D^{Rb}**	-35.0	-35.2	-20.6	-19.9	-43.7	-31.7	**-40.3**
**E^{Rb}**	-35.7	-34.6	-14.2	-12.4	-40.4	-30.6	**-35.3**
**$TS(E\text{-}F)^{Rb}$**	-18.6	-21.5	5.5	5.4	-31.6	-12.5	**-25.5**
**F^{Rb}**	-36.6	-37.5	-17.5	-18.6	-43.1	-33.6	**-40.1**
**$TS(F\text{-}G)^{Rb}$**	-31.1	-32.3	-4.6	-4.0	-43.8	-26.4	**-39.1**
**G^{Rb}**	-61.6	-62.4	-34.6	-36.8	-76.5	-56.4	**-71.2**
**A^{Cs}**	0.0	0.0	0.0	0.0	0.0	0.0	**0.0**
**B^{Cs}**	-0.9	0.2	6.7	7.0	2.6	0.5	**4.1**
**$TS(B\text{-}C)^{Cs}$**	15.9	13.4	25.9	26.5	8.5	18.9	**11.5**
**C^{Cs}**	2.6	1.9	9.6	8.6	-2.7	2.7	**-2.6**
**$TS(C\text{-}D)^{Cs}$**	3.1	2.0	14.0	13.9	-2.2	4.0	**-1.2**
**D^{Cs}**	-36.1	-36.2	-21.8	-21.1	-43.3	-32.7	**-40.0**
**E^{Cs}**	-36.8	-35.6	-13.5	-12.3	-40.7	-31.9	**-35.8**
**$TS(E\text{-}F)^{Cs}$**	-19.8	-22.4	5.5	6.6	-33.1	-13.3	**-26.6**
**F^{Cs}**	-33.4	-34.0	-9.5	-9.8	-43.1	-29.1	**-38.8**
**$TS(F\text{-}G)^{Cs}$**	-32.6	-33.7	-6.7	-6.3	-42.7	-28.0	**-38.1**
**G^{Cs}**	-71.8	-72.0	-42.0	-40.8	-85.5	-64.9	**-78.6**

References

1. Dolomanov, O. V.; Bourhis, L. J.; Gildea, R. J.; Howard, J. A. K.; Puschmann, H., *J. Appl. Cryst.* **2009**, *42*, 339-341.
2. Sheldrick, G. M., *Acta Cryst*. **2015**, *A71*, 3-8.
3. Sheldrick, G. M., *Acta Cryst*. **2015**, *C71*, 3-8.
4. M. J. Frisch, G. W. Trucks, H. B. Schlegel, G. E. Scuseria, M. A. Robb, J. R. Cheeseman, G. Scalmani, V. Barone, G. A. Petersson, H. Nakatsuji, X. Li, M. Caricato, A. V. Marenich, J. Bloino, B. G. Janesko, R. Gomperts, B. Mennucci, H. P. Hratchian, J. V. Ortiz, A. F. Izmaylov, J. L. Sonnenberg, Williams, F. Ding, F. Lipparini, F. Egidi, J. Goings, B. Peng, A. Petrone, T. Henderson, D. Ranasinghe, V. G. Zakrzewski, J. Gao, N. Rega, G. Zheng, W. Liang, M. Hada, M. Ehara, K. Toyota, R. Fukuda, J. Hasegawa, M. Ishida, T. Nakajima, Y. Honda, O. Kitao, H. Nakai, T. Vreven, K. Throssell, J. A. Montgomery Jr., J. E. Peralta, F. Ogliaro, M. J. Bearpark, J. J. Heyd, E. N. Brothers, K. N. Kudin, V. N. Staroverov, T. A. Keith, R. Kobayashi, J. Normand, K. Raghavachari, A. P. Rendell, J. C. Burant, S. S. Iyengar, J. Tomasi, M. Cossi, J. M. Millam, M. Klene, C. Adamo, R. Cammi, J. W. Ochterski, R. L. Martin, K. Morokuma, O. Farkas, J. B. Foresman, D. J. Fox, Wallingford, CT, 2016.
5. Andrae, D.; Häußermann, U.; Dolg, M.; Stoll, H.; Preuß, H. T*heor. Chim. Acta* **1990**, *77*, 123-141.
6. (a) Hariharan, P. C.; Pople, J. A. *Theor. Chim. Acta* **1973**, *28*, 213-222; (b) Hehre, W. J.; Ditchfield, R.; Pople, J. A.; *J. Chem. Phys.* **1972**, *56*, 2257-2261.
7. (a) Becke, A. D. *Phys. Rev. A* **1988**, *38*, 3098-3100; (b) Perdew, J. P. *Phys. Rev. B* **1986**, *33*, 8822-8824.
8. Tomasi, J.; Mennucci, B.; Cammi, R. *Chem. Rev.* **2005**, *105*, 2999-3094.
9. Grimme, S.; Ehrlich, S.; Goerigk, L. *J. Comp. Chem.* **2011**, *32*, 1456-1465.
10. Liu, H.-Y.; Hill, M. S.; Mahon, M. F.; McMullin, C. L.; Schwamm, R. J., Seven-Membered Cyclic Diamidoalumanyls of Heavier Alkali Metals: Structures and C–H Activation of Arenes. *Organometallics* **2023,** *42* (19), 2881-2892.
